# Expectation Value-pCCD-Based Methods for Single-Electron Properties

**DOI:** 10.1021/acs.jpca.5c03859

**Published:** 2025-07-16

**Authors:** Rahul Chakraborty, Somayeh Ahmadkhani, Julian Świerczyński, Katharina Boguslawski, Paweł Tecmer

**Affiliations:** Institute of Physics, Faculty of Physics, Astronomy, and Informatics, Nicolaus Copernicus University in Toruń, Grudziadzka 5, Toruń 87-100, Poland

## Abstract

Expectation-value-coupled cluster theory (XCC) offers a simple avenue for molecular property evaluation. However, its potential has not been fully explored for the new computationally inexpensive CC models, such as pair-coupled cluster doubles (pCCD) and post-pCCD extensions. To that end, we implemented and explored one-electron reduced density matrices in the explicitly connected commutator expansion of the expectation value framework [*J. Chem. Phys.* **2006**, 125, 184109] using pCCD, frozen pair Coupled Cluster (fpCC), and frozen pair linearized Coupled Cluster (fpLCC) variants. The expectation-value-based density matrices are calculated directly using the cluster amplitudes and are computationally cheaper than the corresponding response CC densities, as we bypass solving the computationally expensive Λ-equations. The performance of this approach, when combined with the pCCD-based methods, is assessed against the dipole and quadrupole moments of molecules of a varying chemical nature. We benchmarked our results against the response of CCSD­(T) using Hartree–Fock canonical orbitals and variationally optimized pCCD orbitals. Our study highlights that localized pCCD orbitals are a good choice for computing one-electron properties of organic molecules.

## Graphical abstract

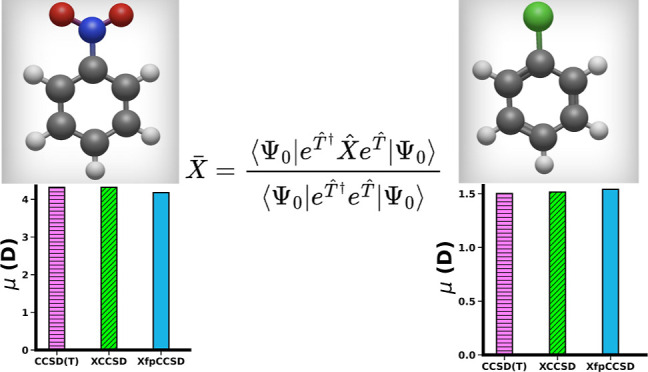

## 1 Introduction

In the last decades, coupled cluster (CC) theory^1–6^ has become the standard tool for wave function theory (WFT)-based quantum chemical studies.^5,7,8^ Concurrent with the theoretical development of various flavors of CC theory, tremendous progress has also been made toward large-scale applications.^9–13^ In this aspect, CC with domain-based local pair natural orbital (DLPNO), which exploits the localized short–range nature of electron correlation, has been extensively used for large chemical systems.^13–16^ Molecular property calculation with this approach has also been devised.^17^ Still, the performance of standard single-reference CC methods leaves room for improvement in cases of strongly correlated systems.^18,19^ Although multireference CC models have been developed,^6,20,21^ they are computationally demanding and dependent on active space choices.

Customizing the CC operator to specifically focus on static correlation, albeit at the expense of losing some dynamic correlation, has been used to circumvent this problem.^22–24^ The antisymmetric product of the 1-reference orbital geminal (AP1roG) method developed by Limacher et al.^24,25^ and later given the moniker pair coupled cluster doubles (pCCD) by Scuseria and co-workers^26,27^ has shown promise along these lines.^19^ This approach delineates the zero-seniority block of the Hilbert space and thus performs well for static correlation.^28,29^ The method offers features such as polynomial scaling, size consistency, and size extensivity (achieved through a variational orbital optimization protocol^25,30–33^ ). Augmented with various dynamic correlation correction techniques such as frozen-pair coupled cluster (fpCC)^34^ and frozen-pair linearized coupled cluster (fpLCC),^35^ pCCD can treat the electron correlation problem in a balanced way. Through numerous studies, pCCD-based methods have established their reliability for a range of chemical problems, including ground- and excited-state properties.^19,36–48^

The evaluation of molecular properties, especially multipole moments, using CC theory still constitutes a very active field of research. The calculation of properties using any CC wave function, including pCCD and post-pCCD ones, is not straightforward due to the nonlinear relation between the wave function and the cluster amplitudes. The standard approach in this case has been to calculate the properties as a response of any operator *X̂* to external perturbation through CC energy derivatives. A generalized form of the Hellmann–Feynman theorem for nonvariational wave functions provides the so-called Λ̂ operator approach.^49–52^

$${\mathrm{X̅}}_{resp}=\left\langle 0\left|\left(1+\mathrm{\Lambda ̂}\right)\right|e^{-\mathrm{T̂}}\mathrm{X̂}e^{\mathrm{T̂}}|0\right\rangle$$ 1

Here, Λ̂ is a de-excitation operator whose amplitudes are solved as zeroth-order Lagrange multipliers, and *T̂* is the excitation operator. The first-order energy derivative and property calculations using this method are generally known as the linear–response CC method. For example, the dipole moment can be calculated as a response of the atom or molecule to an external electric field. In our previous work, we evaluated the performance of pCCD-based methods in terms of response properties for ground-state electronic dipole moments.^53^ We observed that orbital optimization improves the overall statistics in favor of pCCD and post-pCCD methods by introducing the effect of partial inclusion of singles (since any variational orbital optimization protocol is equivalent to adding unitary single orbital rotations). The linearized CCD, added to the orbital-optimized pCCD wave function (referred to as oo-pCCD-LCCD), showed the best performance and provided satisfactory agreement with a standard CCSD approach. However, the singles from the linearized Baker–Campbell–Haussdorf (BCH) equation were found to be unsatisfactory in this regard. The nature and extent of the correction produced by LCC also depend to a great extent on the quality of the reference-orbital-optimized pCCD wave function. For molecules where the pCCD dipole moments in an optimized-orbital basis are worse than those on a canonical basis, oo-pCCD-LCCD does not produce any significant improvement in dipole moment, whereas oo-pCCD-LCCSD overcompensates the error.

This leaves room for other dynamic correlation correction schemes, especially fpCC, to be tested for their performance, in terms of molecular properties. However, response density matrices are currently not available for the fpCC-type methods. An alternate approach is to use the definition of the expectation value of any observable related to any general operator *X̂* in a state with wave function Ψ,

2 $$\mathrm{X̅}=\frac{\langle \Psi |\mathrm{X̂}\Psi \rangle }{\langle \Psi |\Psi \rangle }$$

If the CC wave function is written as $|\Psi \rangle =e^{\mathrm{T̂}}|\Psi_{0}\rangle$ , where |Ψ_0_⟩ is the reference determinant, the corresponding expectation value expression reads.

3 $$\mathrm{X̅}=\frac{\langle \Psi_{0}|e^{{\mathrm{T̂}}^{†}}\mathrm{X̂}e^{\mathrm{T̂}}|\Psi_{0}\rangle }{\langle \Psi_{0}|e^{{\mathrm{T̂}}^{†}}e^{\mathrm{T̂}}|\Psi_{0}\rangle }$$

A major roadblock to directly implementing eq 3 is the problem with the truncation of the infinite series of commutators produced by the expansion of the de-excitation operator ${\mathrm{T̂}}^{†}$ . In this work, we take the route proposed by Jeziorski and Moszyński,^54^ where they use a different excitation operator *Ŝ* $\left(Ŝ={Ŝ}_{1}+{Ŝ}_{2}+{Ŝ}_{3}+...\right)$ defined as

4 $$e^{Ŝ}|\Psi_{0}\rangle =\frac{e^{{\mathrm{T̂}}^{†}}e^{\mathrm{T̂}}|\Psi_{0}\rangle }{\left\langle \Psi_{0}\left|e^{{\mathrm{T̂}}^{†}}e^{\mathrm{T̂}}\right|\Psi_{0}\right\rangle }$$

The *Ŝ* operator is a connected quantity, as has been shown in ref 47, and satisfies a linear equation consisting of a finite number of multiple commutators of *T̂* and *T̂* ^†^. It is to be noted that both response and expectation value approaches converge to the same full configuration interaction (FCI) limit as higher orders of excitations are included in *T̂* (consequently, in *Ŝ*, in case of the expectation value approach). The connected (thus, size-extensive) nature of the *Ŝ* operator approach has been utilized in a number of works to express CC propagators and calculate various single-electron properties.^55–57^ Specifically, Korona and co-workers have shown that with respect to multipole moments and electrostatic interaction energies, the accuracy of the expectation value approach is close to the response CCSD.^56,58,59^ These works used the conventional CCSD *T̂* operator for truncating *Ŝ*. However, this approach has not been tested with other newly devised CC ansätze methods, such as pCCD and post-pCCD methods. To that end, our work focuses on the performance and reliability of the expectation-value CC approach with pCCD and fpCC operators for truncating *Ŝ*. This provides an alternate route for obtaining density matrices with pCCD and post-pCCD methods and for calculating molecular properties other than the response approach. More importantly, it also provides a way to avoid solving the computationally expensive Λ-equations required to get the density matrices from the response approach (cf. eq 1).

In this work, we have implemented the *Ŝ* operator-based expectation-value CC approach with the pCCD and post-pCCD frameworks in the PyBEST software package.^60,61^ Dipole and quadrupole moments of various sets of molecules, such as main-group diatomics and organic molecules, have been calculated on both canonical and variationally optimized pCCD orbital bases for statistical analysis. Specifically, the efficiency of the density matrices obtained with expectation-value pCCD and fpCC has been assessed and compared with expectation-value CCD and CCSD. We compare the performance of these methods with respect to the standard CCSD­(*T*) relaxed dipole and quadrupole moments.

## 2 Theory

### 2.1 pCCD and Post-pCCD Methods

In the pCCD ansatz, the cluster operator ${\mathrm{T̂}}_{2\left(p\right)}$ contains only the pair-double excitations. The consequent wave function looks as

5 $$|\Psi_{pCCD}\rangle =e^{{\mathrm{T̂}}_{2\left(p\right)}}|\Psi_{0}\rangle$$

6 $${\mathrm{T̂}}_{2\left(p\right)}=\sum_{i}^{occ}\sum_{a}^{virt}t_{i\mathrm{i̅}}^{a\mathrm{a̅}}{â}_{a}^{†}{â}_{\mathrm{a̅}}^{†}{â}_{\mathrm{i̅}}{â}_{i}$$

where ${â}_{q}^{†}$ $\left({â}_{\mathrm{q̅}}^{†}\right)$ and ${â}_{q}$ $\left({â}_{\mathrm{q̅}}\right)$ are the electron creation and annihilation operators for α-spin (β-spin) for the q-th spin orbital, respectively, and |Ψ_0_⟩ is a reference wave function (usually the Hartree–Fock method). As pCCD targets the zero-seniority part of the full wave function, it performs very well in capturing a strong electron correlation of closed-shell electronic structures. When augmented with an orbital optimization protocol, pCCD becomes size-consistent and provides reliable results for strongly correlated systems.^24,62,63^ However, a major portion of dynamic electron correlation is excluded from the pCCD wave function, as it includes only some specific configurations. A path for correcting this shortcoming is to use the tailored CC theory^34,64–72^

7 $$|\Psi_{tCC}\rangle =e^{{\mathrm{T̂}}_{ext}}e^{{\mathrm{T̂}}_{\mathrm{int}}}|\Psi_{0}\rangle$$

which is based on the partitioning of the full cluster operator *T̂* into ${\mathrm{T̂}}_{\mathrm{int}}$ and ${\mathrm{T̂}}_{ext}$ . First, ${\mathrm{T̂}}_{\mathrm{int}}$ , generally derived from a method that describes the strong correlation effect, is applied to the reference wave function |Ψ_0_⟩. Subsequently, ${\mathrm{T̂}}_{ext}$ is applied to this intermediate wave function, with the amplitudes of ${\mathrm{T̂}}_{\mathrm{int}}$ held constant to introduce additional excitations and incorporate dynamic correlation into the expanded wave function. Similarly, the dynamic correlation absent in the pCCD wave function can be accounted for by a posteriori corrections, where single and unpaired double excitations are added to the wave function. One such approach is fpCC, which extends the pCCD reference wave function and adds unpaired doubles (fpCCD) or unpaired doubles along with single excitations (fpCCSD) to |Ψ_pCCD_⟩, keeping the cluster amplitudes associated with pair-doubles excitations frozen^27^

8 $$|\Psi_{fpCC}\rangle =e^{{\mathrm{T̂}}_{ext}}|\Psi_{pCCD}\rangle$$

The two variants of this approach mentioned above are fpCCD, where ${\mathrm{T̂}}_{ext}={\mathrm{T̂}}_{2}^{\prime }$ , and fpCCSD, where ${\mathrm{T̂}}_{ext}={\mathrm{T̂}}_{1}+{\mathrm{T̂}}_{2}^{\prime }$ , with ${\mathrm{T̂}}_{2}^{\prime }$ being the cluster operator for nonpair doubles and ${\mathrm{T̂}}_{1}$ being the same for single excitations. These approaches have been used previously to construct potential energy surfaces and calculate the spectroscopic constants of a range of molecules. Notably, Leszczyk et al.^34^ have shown that fpCCSD performs similarly to CCSD tailored with the density matrix renormalization group (DMRG-tCCSD).

A linearized form of the fpCC, which we denote as fpLCC (also noted as pCCD-LCC in the literature), has also been employed for dynamic correlation correction purposes,

9 $$|\Psi_{fpLCC}\rangle \approx \left(1+{\mathrm{T̂}}_{ext}\right)|\Psi_{pCCD}\rangle$$

In the fpLCC framework,^35^ the associated Baker–Campbell–Hausdorff expansion is restricted to the second term, i.e.,

10 $$\left(Ĥ+\left[Ĥ,{\mathrm{T̂}}^{\prime }\right]\right)|\Psi_{pCCD}\rangle =E|\Psi_{pCCD}\rangle$$

Like fpCC, fpLCC can have only doubles (fpLCCD) or singles and doubles (fpLCCSD) excitation in the model. Though computationally cheaper, fpLCC can exhibit diverging points in PES and numerically unphysical response dipole moments.^34,53^

Table 1 provides a summary of all CC models investigated in this work and the concerned reference wave functions, cluster operators, and computational scalings. Though CCD, CCSD, fpCCD, fpCCSD, fpLCCD, and fpLCCSD all appear to have the same computational scaling, they mostly differ in their convergence, which originates from the inclusion/exclusion of nonlinear terms and/or the choice of the reference determinant (HF or pCCD-optimized orbitals). It is to be noted that though pCCD itself has a scaling of $O\left(o^{2}v^{2}\right)$ , the 4-index transformation of the two-electron repulsion integrals during the orbital optimization procedure takes the scaling to $O\left(N^{5}\right)$ unless the Cholesky decomposition or the density fitting is utilized. A subtle aspect that can be argued here is that orbital-optimized pCCD (and the dynamic correlations thereafter), in a strictly theoretical sense, is no longer a post-HF method, as the MO basis itself is entirely different. The reference Slater determinant is no longer the same as |Ψ_HF_⟩. Still, for the sake of simplicity and keeping in mind that the pCCD orbital optimization process starts from the HF reference, we treat it under the post-HF class of methods.

**Table 1:** Summary of Investigated CC Models^a^

method	CC operator	|Ψ_0_⟩	scaling	memory
CCD	$e^{{\mathrm{T̂}}_{2}}$	|Ψ_HF_⟩	$O\left(o^{2}v^{4}\right)$	$O\left(v^{4}\right)$
CCSD	$e^{{\mathrm{T̂}}_{1}+{\mathrm{T̂}}_{2}}$	|Ψ_HF_⟩	$O\left(o^{2}v^{4}\right)$	$O\left(v^{4}\right)$
pCCD	$e^{{\mathrm{T̂}}_{2\left(p\right)}}$	|Ψ_SD_⟩	$O\left(o^{2}v^{2}\right)$	$O\left(N^{3}\right)$
fpCCD	$e^{{\mathrm{T̂}}_{2}^{\prime }}$	|Ψ_pCCD_⟩	$O\left(o^{2}v^{4}\right)$	$O\left(v^{4}\right)$
fpCCSD	$e^{{\mathrm{T̂}}_{1}+{\mathrm{T̂}}_{2}^{\prime }}$	|Ψ_pCCD_⟩	$O\left(o^{2}v^{4}\right)$	$O\left(v^{4}\right)$
fpLCCD	1+ ${\mathrm{T̂}}_{2}^{\prime }$	|Ψ_pCCD_⟩	$$O\left(o^{2}v^{4}\right)$$	$$O\left(v^{4}\right)$$
fpLCCSD	1+ ${\mathrm{T̂}}_{1}$ + ${\mathrm{T̂}}_{2}^{\prime }$	|Ψ_pCCD_⟩	$$O\left(o^{2}v^{4}\right)$$	$$O\left(v^{4}\right)$$

a Reference wave functions (Ψ_0_), the form of the CC operator, and formal computational scalings of all the different flavors of CC studied in this work. *o* and *v* denote the number of occupied and virtual molecular orbitals, respectively, and *N* = o + v. ${\mathrm{T̂}}_{2}^{\prime }$ is a doubles operator for unpaired excitation, that is ${\mathrm{T̂}}_{2}^{\prime }={\mathrm{T̂}}_{2}-{\mathrm{T̂}}_{2\left(p\right)}$ . Note that we employ a single Slater determinant reference function for pCCD constructed from the optimized pCCD natural orbitals, labeled as |Ψ_SD_⟩. Memory indicates the order of the storage bottleneck (excluding a solver-dependent pre-factor).

A separate important question regarding the property calculation is the difference in computational complexity between the expectation-value method and the response approach. The response approach brings in the additional cost of solving the response Λ-equations. This procedure has the same scaling as solving the amplitude equation, that is, $O\left(o^{2}v^{4}\right)$ . The expectation-value approach entails no additional computational burden, as the equations involve already-calculated CC amplitudes. For orbital-optimized pCCD, the Λ-equations are solved anyway during the orbital optimization process, and hence, the response property calculations do not require any additional cost. However, the same is not true for fpCC and fpLCC. A direct comparison can be made for fpLCC, for which the response framework was previously implemented in PyBEST. For nitrobenzene, our largest test molecule with 261 contracted basis functions and 23 active occupied and 229 active unoccupied orbitals, the Λ-equations for the fpLCCD and fpLCCSD methods took ∼103 and ∼204 min, respectively (8 CPUs on Intel­(R) Xeon­(R) Gold 6336Y CPU at 2.40 GHz)

### 2.2 Expectation-Value-Based Density Matrices from pCCD and Related Methods

The particular form of the expectation CC model implemented in this work closely follows previous works by Korona.^59^ Combining eqs 3 and 4, the average value of an operator can be written as

11 $$\mathrm{X̅}=\langle e^{Ŝ}|e^{-\mathrm{T̂}}\mathrm{X̂}e^{\mathrm{T̂}}\rangle =\left\langle e^{{Ŝ}^{†}}e^{-\mathrm{T̂}}\mathrm{X̂}e^{\mathrm{T̂}}e^{-{Ŝ}^{†}}\right\rangle$$

This equation can then be rewritten with nested commutators using the BCH formulation

12 $$e^{-Ŷ}\mathrm{X̂}e^{Ŷ}=\mathrm{X̂}+\left[\mathrm{X̂},Ŷ\right]+\frac{1}{2!}\left[\left[\mathrm{X̂},Ŷ\right],Ŷ\right]+...$$

where $Ŷ=-\mathrm{T̂}{Ŝ}^{†}$ .

Following Jeziorski and Moszyński,^54^ *Ŝ* is expanded (*S* = *S* ^[1]^ + *S* ^[2]^ +··· *S* ^[*k*]^, where k is the highest level of electron excitation in *T̂*) in terms of *T̂* and ${\mathrm{T̂}}^{†}$ . If *T̂* is truncated at the CCSD excitation level, then we arrive at the following equation

13 X̅=⟨X̂⟩+⟨Ŝ1|X̂⟩+⟨X̂|T̂1⟩+⟨Ŝ2|[X̂,T̂2]⟩+⟨Ŝ1|[X̂,T̂2]⟩+⟨Ŝ1|[X̂,T̂1]⟩+⟨Ŝ2|[[X̂,T̂1],T̂2]⟩+12⟨Ŝ12|[X̂,T̂2]⟩+12⟨Ŝ1Ŝ2|[[X̂,T̂2],T̂2]⟩+12⟨Ŝ1|[[X̂,T̂1],T̂1]⟩+12⟨Ŝ3|[[X̂,T̂2],T̂2]⟩+12⟨Ŝ12|[[X̂,T̂1],T̂2]⟩+112⟨Ŝ13|[[X̂,T̂2],T̂2]⟩

Ignoring terms higher than $O\left({\mathrm{T̂}}^{2}\right)$ and with an additional omission of $\left\langle [{Ŝ}_{1}^{\left[1\right]†},\left[\mathrm{X̂},{\mathrm{T̂}}_{1}\right]\right\rangle$ , we get

14 X̅=⟨X̂⟩+⟨[Ŝ1[1]†,X̂]⟩+⟨[Ŝ1[2]†,X̂]⟩+⟨[X̂,T̂1]⟩+⟨[Ŝ2[1]†,[X̂,T̂2]]⟩+⟨[Ŝ1[1]†,[X̂,T̂2]]⟩.

The *Ŝ* ^[*n*]^ terms are given as

15 $${Ŝ}_{1}^{\left[1\right]}={\mathrm{T̂}}_{1}$$

16 $${Ŝ}_{2}^{\left[1\right]}={\mathrm{T̂}}_{2}$$

17 $${Ŝ}_{1}^{\left[2\right]}={\mathrm{P̂}}_{1}\left([{\mathrm{T̂}}_{1}^{†},{\mathrm{T̂}}_{2}]\right)$$

where ${\mathrm{P̂}}_{n}\left(\mathrm{X̂}\right)={\left(n!\right)}^{-2}\sum_{i_{1}...i_{n}}\sum_{a_{1}...a_{n}}\left\langle {â}_{a_{1}}{â}_{i_{1}}^{†}...{â}_{a_{n}}{â}_{i_{n}}^{†}\mathrm{X̂}\right\rangle {â}_{a_{1}}^{†}{â}_{i_{1}}...{â}_{a_{n}}^{†}{â}_{i_{n}}$ is the superoperator projecting on the space spanned by *n*-tuple excitation operators. This approximation has been labeled as XCCSD(3) (due to third-order Møller–Plesset terms in the equation). It was shown that the differences between the results produced by full XCCSD and the approximated XCCSD(3) models are insignificant due to the low contribution of the higher-order terms in eq 13. For simplicity, we do not distinguish between the two approaches, and henceforth, we refer to the approximation as the XCCSD.

The correlation contribution to the one-particle reduced density matrix γ can be derived from eq 14 by placing $\mathrm{X̂}=E_{p}^{q}$ , where *E*_p_^q^ is the singlet excitation operator.

$$E_{p}^{q}={â}_{q}^{†}{â}_{p}+{â}_{\mathrm{q̅}}^{†}{â}_{\mathrm{p̅}}$$ 18

The different blocks of γ are then given as

19 $$occupied-virtual:\gamma_{i}^{a}=2\left(t_{i}^{a}+\sum_{jb}t_{i}^{a}t_{-ij}^{ab}\right)=\gamma_{a}^{i}$$

20 $$occupied-occupied:\gamma_{i}^{k}=-\sum_{jab}t_{ab}^{ij}t_{ab}^{-kj}$$

21 $$virtual-virtual:\gamma_{c}^{a}=-\sum_{ijb}t_{ab}^{ij}t_{cb}^{-ij}$$

where $t_{-ij}^{ab}=2t_{ij}^{ab}-t_{ij}^{ba}$ and *t*_*i.*._^a..^ are the spin-free amplitudes of single, double, etc. excitations.

In this study, we obtain the spin-free single amplitudes *t*_*i*_^a^ and double amplitudes *t*_*ij*_^ab^ from the various models discussed earlier. These amplitudes are then used in eqs 19–21 to construct the corresponding density matrices of a given CC model. Like XCCSD, we label the expectation-value-based approaches as XpCCD, XfpCC, and XfpLCC. XfpCC and XfpLCC have two flavors as discussed in Section 2.1 based on the inclusion of singles excitation. We have also investigated the density matrices obtained using the expectation-value framework for the CCD method, i.e., XCCD. For XpCCD, XCCD, XfpCCD, and XfpLCCD, the *γ*_i_^a^ terms related to the occupied-virtual block of the density matrix are 0. Dipole and quadrupole moments are computed accordingly after the density matrix.

## 3 Computational Details

### 3.1 Calculation Protocols

The axial components of dipole and quadrupole moments are given as

22 $$\mu_{\alpha }=-\int \rho \left(r\right)r_{\alpha }dV+\sum_{i=1}^{N_{i}}Z_{i}R_{i\alpha }$$

and

23 $$Q_{\alpha \beta }=-\frac{1}{2}\int \rho \left(r\right)\left(3r_{\alpha }r_{\beta }-\delta_{\alpha \beta }r^{2}\right)dV+\frac{1}{2}\sum_{i=1}^{N_{i}}Z_{i}\left(3R_{i\alpha }R_{i\beta }-\delta_{\alpha \beta }R_{i}^{2}\right)$$

respectively, where α and β denote the Cartesian axes, ρ is the electron density, *Z* is the nuclear charge, and *r* and *R* are the electronic and nuclear coordinates, respectively. The quadrupole moment also forms a traceless tensor (*Q*_*xx*_ + *Q*_*yy*_ + *Q*_*zz*_ = 0). For simplicity, we consider the *Q*_*zz*_ component for comparison.

After introducing an atomic orbital (AO) basis set, one α-component of the dipole moment is evaluated from

$$\mu_{\alpha }=\overset{N_{i}}{\underset{i=1}{\sum }}Z_{i}R_{i\alpha }-\underset{\mu }{\sum }\underset{\nu }{\sum }\gamma_{\nu }^{\mu }\langle \nu |r_{\alpha }|\mu \rangle$$ 24

and the two components of the quadrupole moment from

$$Q_{\alpha \beta }=\overset{N_{i}}{\underset{i=1}{\sum }}\frac{3}{2}Z_{i}R_{i\alpha \beta }-\underset{\mu }{\sum }\underset{\nu }{\sum }\gamma_{\nu }^{\mu }\langle \nu |q_{\alpha \beta }|\mu \rangle$$ 25

$$Q_{\alpha \alpha }=\overset{N_{i}}{\underset{i=1}{\sum }}\frac{1}{2}Z_{i}\left(3R_{i\alpha \alpha }-R_{rr}\right)\underset{\mu }{\sum }\underset{\nu }{\sum }\gamma_{\nu }^{\mu }\langle \nu |q_{\alpha \alpha }|\mu \rangle$$ 26

where γ_ν_^μ^ is the density matrix in the AO basis, and $\langle \nu |{\mathrm{r̂}}_{\alpha }|\mu \rangle =\int \chi_{\nu }^{*}\left(r\right)r_{\alpha }\chi_{\mu }\left(r\right)dr$ and $\langle \nu |q_{\alpha \beta }|\mu \rangle =\int \chi_{\nu }^{*}\left(r\right)q_{\alpha \beta }\chi_{\mu }\left(r\right)dr$ are the dipole and quadrupole moment integrals, respectively, expressed in the AO basis {χ_ν_},^74^ while *R*_*rr*_ = *R*_*xx*_ + *R*_*yy*_ + *R*_*zz*_. Since all pCCD-based methods work in the molecular orbital (MO) basis and hence the corresponding 1-RDMs are defined for the molecular orbitals, we need to perform an AO–MO transformation step of the dipole moment integrals or the 1-RDMs, respectively.

All expectation value CC approach-based calculations, with XpCCD, XCC, XfpCC, and XfpLCC methods, were implemented and performed in the developer version (v1.4.0-dev) of the **PyBEST** software package.^60,61,75^ The dipole and traceless quadrupole moments were calculated from the expectation value 1-RDM (γ_ν_^μ^) using the Sadlej-pVTZ basis set, which is specifically designed for efficient description of electronic properties^76,77^ and shows comparable accuracy as aug-cc-pVTZ.^78^ For the dipole moment surface (DMS), we have used the cc-pVDZ basis set to be able to compare our data with the reference FCI values available in the literature. For the potential dipole moment surface of the HF molecule, we also used a cc-pVDZ basis set to compare our data with the reference FCI values. In this work, we used two different molecular orbital bases, i.e., the canonical HF orbitals and the pCCD-optimized orbitals. Henceforth, all calculations with canonical HF orbitals will be marked as Method­(HF), and the same calculations with pCCD optimized orbitals will be marked as Method­(OO). Cholesky decomposition with a threshold of 10^–5^ was used in all calculations except for LiF and LiNa, where we faced convergence issues at the SCF level. Pipek–Mezey orbital localization^79^ was used to speed up the orbital optimization process. All nonvalence orbitals were kept frozen for all post-HF calculations. Orbital relaxed CCSD and CCSD­(T) response dipole and quadrupole moment values using canonical HF molecular orbitals were obtained from the MOLPRO2019 package.^80–82^ The Supporting Information gives all of the calculated total dipole moment and quadrupole moment zz-component values.

### 3.2 Test Set of Molecules

We selected 50 molecules covering a wide range of chemical characteristics. Of these, 28 are small molecules (listed in Table 2), including main-group diatomics and small polyatomic inorganic compounds and ions, while 22 are organic molecules (structures shown in Figure 1). Among these, 25 small and 20 organic molecules exhibit a dipole moment. The bond lengths of the main group diatomic molecules were taken from Liu et al.^83^ and references therein. Coordinates of the larger molecules were taken from Jensen et al.^84^ Each diatomic molecule is placed along the *z*-axis. For some organic molecules (acetaldehyde, acetone, 3-pentanone, *N*-methylformamide, propanamide, propanoic acid, cyclopentane, chlorobenzene, and nitrobenzene), the axes were changed in such a way that the largest absolute component of the traceless quadrupole moment always lay on the *z*-axis. The coordinates of all molecules are provided in the Supporting Information.

**Table 2:** List of Chemical Species Included in the “Small” Set in This Work.^a^

diatomic	polyatomic	ionic
H_2_, LiH, NaH, LiF, NaF, NaCl, HF, HCl, LiNa, ClF, N_2_, PN, CO, CS, CSe, SiO, SiS, SiSe, GeO, GeS, GeSe	H_2_O, CO_2_, NH_3_, PH_3_	OH, NO^+^, CN^–^

a The structural parameters are given in the Supporting Information.

**Figure 1:**
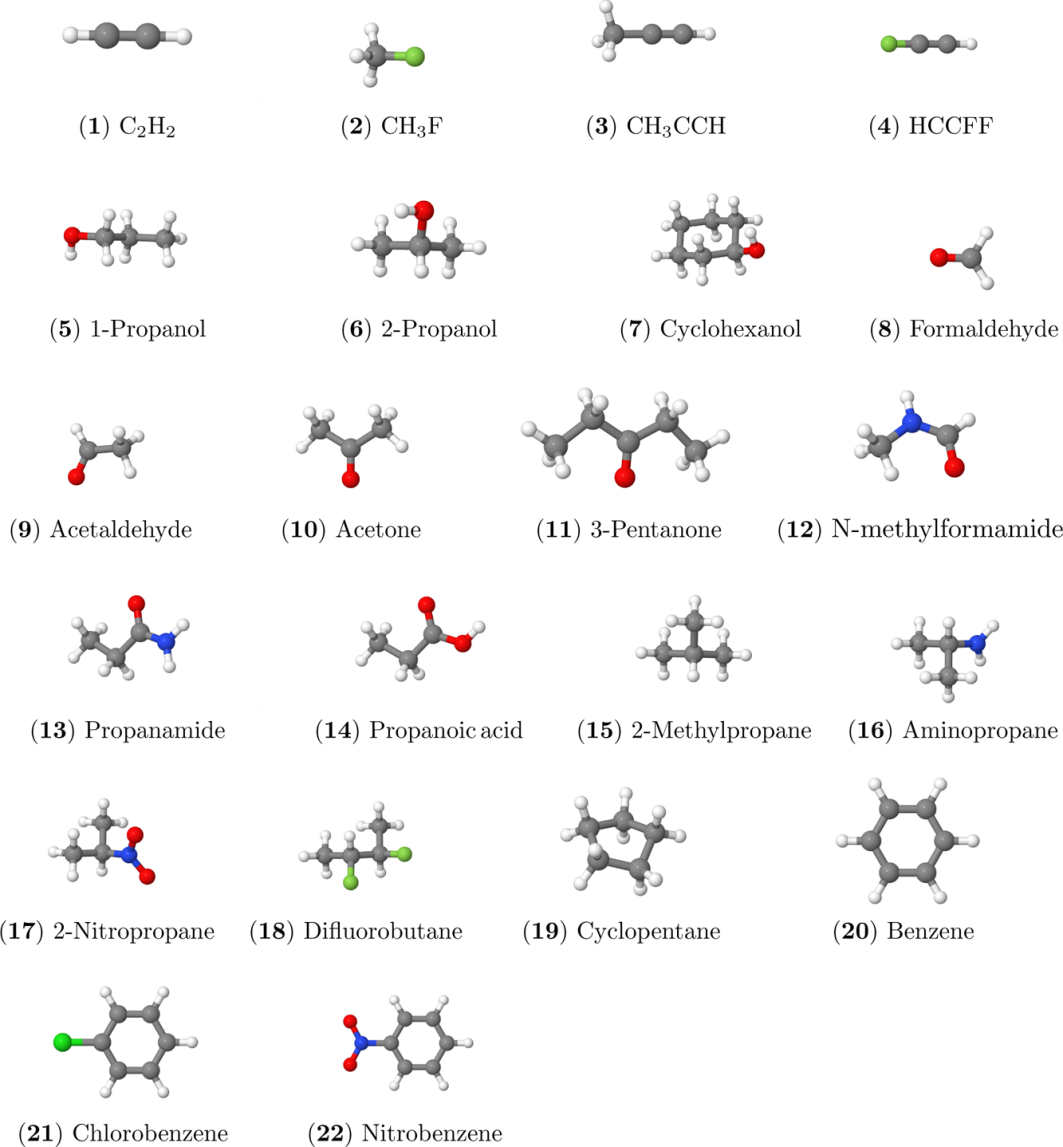
Molecular structures of organic molecules investigated in this work. Coordinates are given in the Supporting Information. The molecular structures were drawn using the Jmol software package.^73^

## 4 Results and Discussion

### 4.1 Dipole Moment

We start by assessing the accuracy of our dipole moments computed with various pCCD-based methods for different molecular test sets at their equilibrium geometries (Subsection [Sec sec3.2]). Specifically, we focus on the statistical parameters obtained for the “full set” (combining “small” and “organic” sets) to draw an overall picture. A detailed analysis of the different classes of compounds will be done in Subsection [Sec sec4.1.1]. Figures 2 and 3 show the signed errors calculated against orbital relaxed CCSD­(T) response dipole moment values (μ_Method_ – μ_CCSD(T)_) for individual chemical species for the sets of “small” and “organic”, respectively. The corresponding dipole moment numerical values are collected in the Supporting Information. Here, a positive error indicates an overestimation of μ, whereas a negative error means vice versa. Additionally, Table S1 of the Supporting Information summarizes the statistical parameters for all the methods calculated against the orbital relaxed CCSD response dipole moment values. In our statistical analysis for dipole moment, we have excluded the most problematic molecules, CO and SiSe, as they showed abruptly high relative errors ((μ_Method_ – μ_CCSD(T)_)/μ_CCSD(T)_) for some expectation-value-based methods, which leads to ambiguous statistical parameters. For example, XCCSD shows 119% and 116% relative errors for CO, in canonical and pCCD-optimized orbital basis, respectively, with respect to CCSD­(T). SiSe, on the other hand, shows a relative error of 97% and 91% for XpCCD and XfpCCD with pCCD-optimized orbitals. It has been discussed in previous works that for diatomics of group 16 atoms, full triples treatment is required for a reliable prediction of the dipole moment.^59,85^ For such molecules, even the inclusion of perturbative triples may not be sufficient to produce reliable dipole moment values. Hence, particularly for these molecules, CCSD­(T) is not a suitable reference.

**Figure 2:**
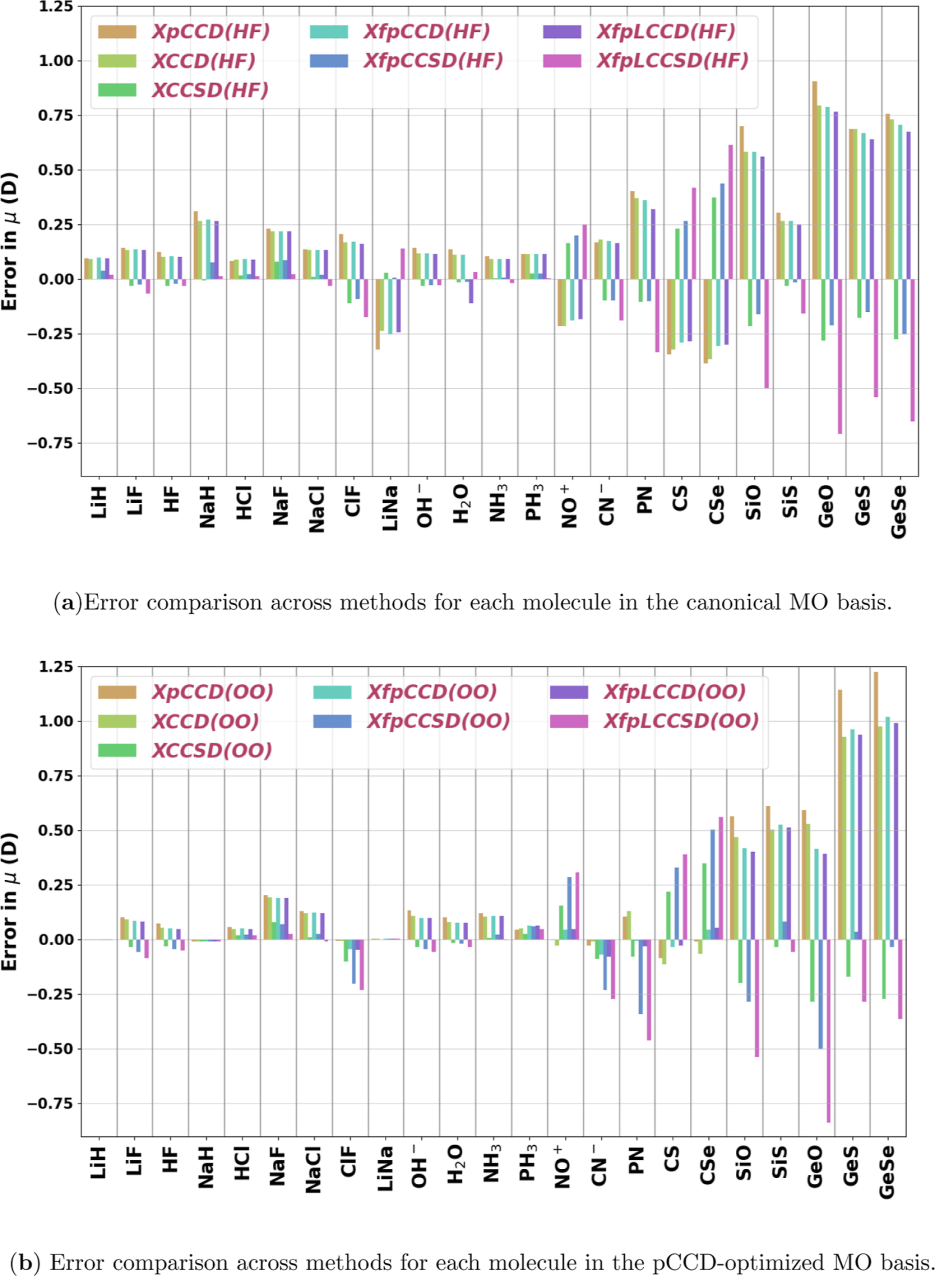
Signed errors with respect to CCSD­(T) (μ_Method_ – μ_CCSD(T)_) in total dipole moments (D) for all methods using the Sadlej-pVTZ basis set for the set of small molecules investigated in this work. (HF) in (a) and (OO) in (b) denote the use of canonical and pCCD-optimized molecular orbitals, respectively.

**Figure 3:**
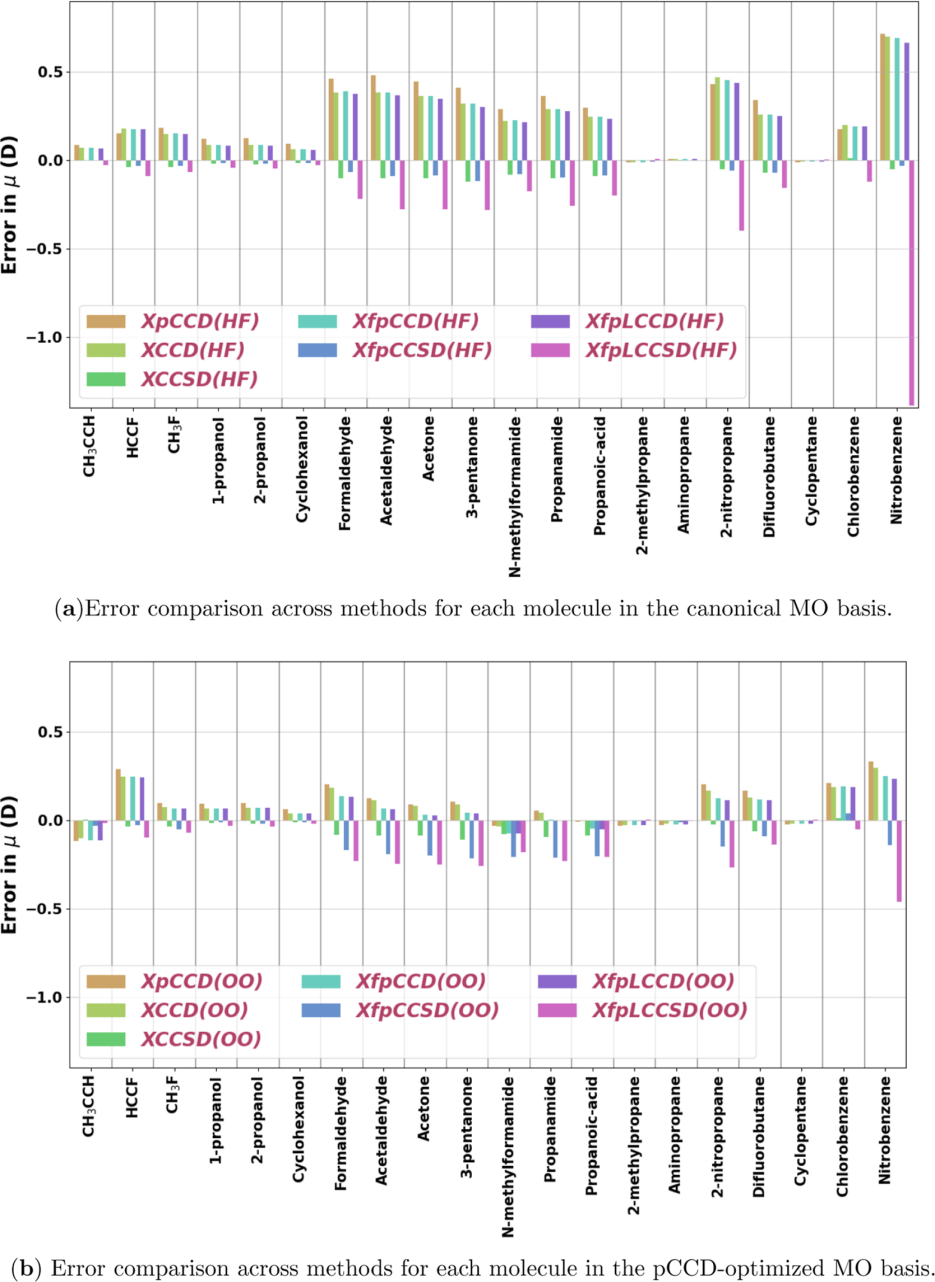
Signed errors with respect to CCSD­(T) (μ_Method_ – μ_CCSD(T)_) in total dipole moments (D) for all methods using the Sadlej-pVTZ basis set for the set of organic molecules investigated in this work. (HF) in (a) and (OO) in (b) denote the use of canonical and pCCD-optimized molecular orbitals, respectively.

Table 3 lists the statistical parameters with respect to the response CCSD­(T) dipole moments for each of the methods in the two molecular orbital bases. Figure 4 illustrates the comparison of the RMSE for all of the methods. It is evident from Figure 4 that XCCSD produces the lowest statistical error parameters. XCCSD is also agnostic to the molecular orbital basis as its performance improves only to a small extent when pCCD-optimized orbitals are used. XfpCCSD shows the best performance among the frozen pair methods. Employing the canonical HF orbital basis, it approaches the accuracy of XCCSD. For all the methods, the statistical parameters for the “full set” improve upon using pCCD-optimized orbitals, except for fpCCSD. The role of orbital rotations, which act like pseudosingles excitations in the wave function, is prominent when we look at the significant improvements in the statistics for the methods containing only double excitations in the cluster operator, i.e., XCCD, XfpCCD, and XfpLCCD. This indicates an overcorrection due to singles excitation in the case of fpCCSD comes from both orbital rotations during orbital optimization and from the inclusion of ${\mathrm{T̂}}_{1}$ in ${\mathrm{T̂}}_{fpCC}$ . The “correction” effect of adding single excitations on the dipole moment is also prominent when we look at the opposite signs of ME for XCCD and XCCSD, or for XfpCCD and XfpCCSD. Both the doubles-only methods, XCCD and XfpCCD, overestimate the dipole moments (+ sign of ME), whereas XCCSD and XfpCCSD underestimate on average with the – sign of ME. In both cases, the values of RMSE, MAE, and MAPE also improve upon adding single excitations to the method. Hence, for both XCC and XfpCC, adding single excitations in the cluster operator takes the dipole moment values closer to the reference. However, the same does not seem to be true between fpLCCD and fpLCCSD. Introducing singles in the linearized version of the fpCC model worsens the RMSE. Interestingly, XfpLCCSD shows lower MAPE than XfpLCCD. This indicates that the performance of XfpLCCSD becomes worse compared to that of XfpLCCD primarily for molecules with a high magnitude of dipole moment.

**Table 3:** Statistical Analysis of Dipole Moments (μ in D) from Various CC- and pCCD-Based Methods w.r.t. Orbital Relaxed Response CCSD­(T) Data Using the Sadlej-pVTZ Basis Set

method	RMSE (D)			ME (D)			MAE (D)			MAPE		
	full set	small	organic	full set	small	organic	full set	small	organic	full set	small	organic
XpCCD(HF)	0.356	0.383	0.323	0.225	0.195	0.259	0.285	0.305	0.261	14.9	16.4	13.2
XpCCD(OO)	0.321	0.417	0.149	0.163	0.221	0.096	0.180	0.232	0.119	12.9	13.8	11.8
XCCD(HF)	0.322	0.351	0.285	0.200	0.180	0.224	0.254	0.279	0.225	13.3	14.8	11.4
XCCD(OO)	0.265	0.343	0.127	0.134	0.181	0.080	0.154	0.201	0.100	10.9	11.8	10.0
XCCSD(HF)	0.116	0.147	0.065	–0.034	–0.020	–0.049	0.078	0.102	0.051	3.8	5.2	2.2
XCCSD(OO)	0.109	0.140	0.056	–0.030	–0.021	–0.040	0.071	0.096	0.042	3.6	4.9	2.0
XfpCCD(HF)	0.316	0.342	0.283	0.201	0.182	0.223	0.250	0.272	0.225	13.1	14.6	11.4
XfpCCD(OO)	0.262	0.343	0.113	0.123	0.179	0.059	0.145	0.193	0.089	10.8	11.8	9.7
XfpCCSD(HF)	0.116	0.149	0.058	–0.020	0.001	–0.044	0.075	0.102	0.044	3.6	5.0	2.0
XfpCCSD(OO)	0.177	0.210	0.129	–0.051	–0.014	–0.095	0.120	0.139	0.098	5.8	7.0	4.4
XfpLCCD(HF)	0.304	0.328	0.273	0.193	–0.173	0.214	0.241	0.262	0.216	12.6	14.0	11.1
XfpLCCD(OO)	0.255	0.333	0.109	0.118	0.174	0.055	0.142	0.191	0.085	10.7	11.6	9.6
XfpLCCSD(HF)	0.337	0.318	0.357	–0.139	0.085	–0.201	0.210	0.216	0.203	9.8	11.1	8.3
XfpLCCSD(OO)	0.256	0.304	0.186	–0.109	–0.084	–0.139	0.173	0.202	0.140	8.4	9.8	6.7

**Figure 4:**
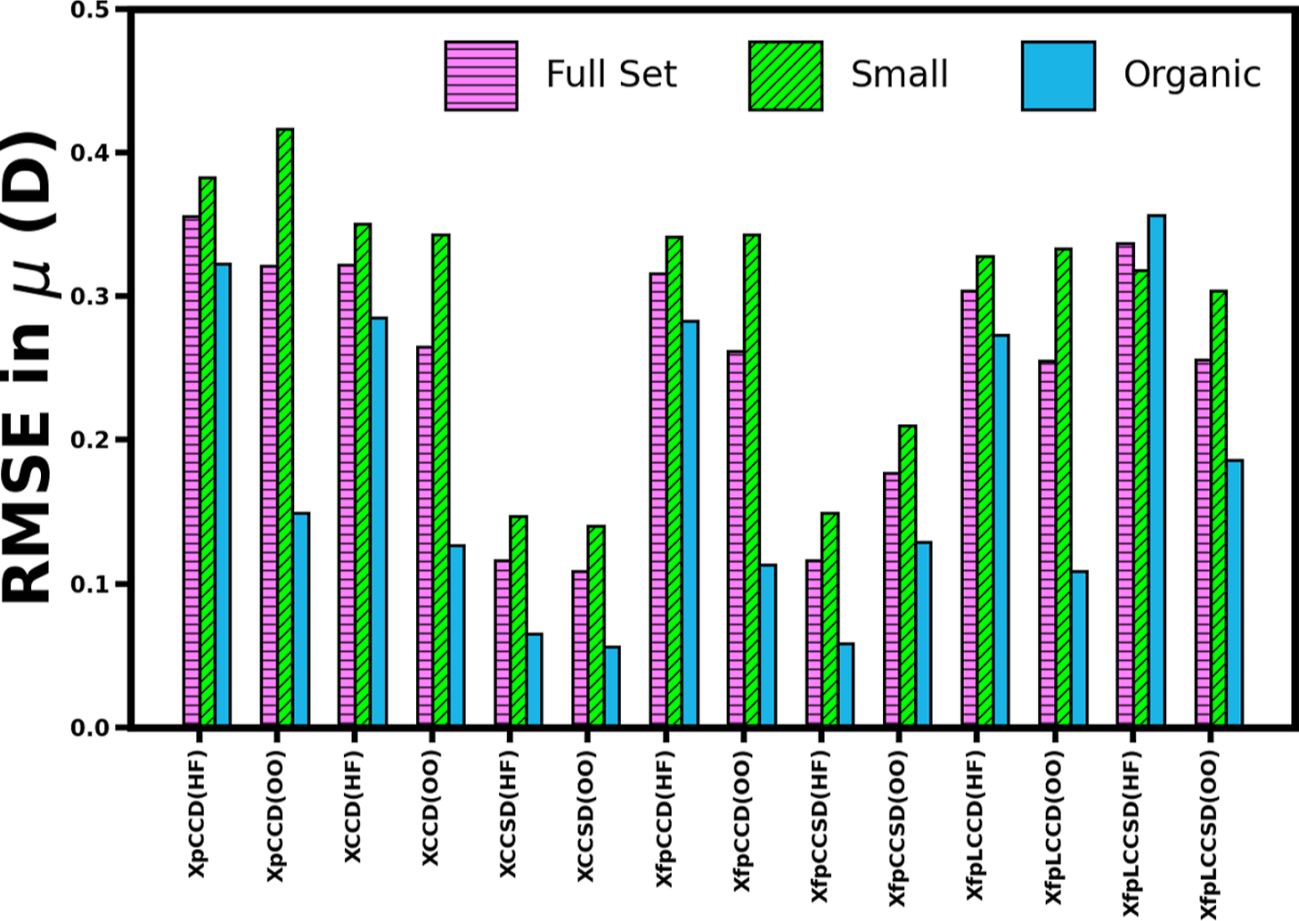
Comparison of CC- and pCCD-based root-mean-square-error (RMSE) of dipole moments (μ in D) w.r.t. response CCSD­(T). RMSE is calculated as $\sqrt{\left(\sum_{i}^{N}{\left(\mu_{Method,i}-\mu_{CCSD\left(T\right),i}\right)}^{2}\right)/N})$ . The sets of molecules have been described in Section 3.2. (HF) and (OO) denote the use of canonical and pCCD-optimized molecular orbitals, respectively. CO and SiSe have been excluded from the analysis.

The statistical measures include the root-mean-square-error (RMSE): $\sqrt{\left(\sum_{i}^{N}{\left(\mu_{Method,i}-\mu_{CCSD\left(T\right),i}\right)}^{2}\right)/N})$ , mean error (ME): $\sum_{i}^{N}\left(\mu_{Method,i}-\mu_{CCSD\left(T\right),i}\right)$ )/*N*, mean-absolute-error (MAE): $\sum_{i}^{N}\left|\left(\mu_{Method,i}-\mu_{CCSD\left(T\right),i}\right)\right|$ )/*N*, and mean-absolute-percentage-error (MAPE): ${\overset{N}{\underset{i}{\sum }}[\left|\left(\mu_{Method,i}-\mu_{CCSD\left(T\right),i}\right)\right|/(\mu_{CCSD\left(T\right),i}]}^{*}100/N$ . The molecular sets are described in Section 3.2. (HF) and (OO) denote the use of canonical and pCCD-optimized orbitals, respectively. CO and SiSe are excluded from the analysis.

#### 4.1.1 Analysis for Separate Molecule Sets

A key aspect of this analysis is to answer how the methods behave for the different molecule classes, i.e., for the set of diatomics, small inorganic polyatomics, and larger organic molecules. A quick look at the statistical averages shows the different behaviors of the methods for the two molecular classes. Figure 4 shows that for all the methods, the RMSE values are significantly lower for organic molecules. For the set of “small” molecules, the transition from canonical to pCCD-optimized orbitals worsens the RMSE values of XpCCD, XfpCCSD, and XfpLCCD. For other methods, it remains stagnant; that is, it has almost no or small improvement with pCCD-optimized orbitals in the case of the small molecules. That is also true for the other statistical parameters presented in Table 3. That motivates an in-depth inspection of the performance of the methods for the different molecular classes taken in this work.

In Figure 5, we show the distribution of the signed percentage errors for (a) small molecules and ions and (b) organic molecules, respectively. For quantities like dipole and quadrupole moments, which can have a very small magnitude for some molecules, the percentage error provides a better assessment tool rather than a simple difference from the reference. In the case of small molecules (Figure 5a), most methods predict the median as positive or very close to zero, except for XfpLCCD, which shows a negative median. The distribution of errors for XCCSD and XfpCCSD using the canonical orbital basis is very similar. It shows that the similar performance between these two methods is not only a coincidence limited to statistical averaging but a molecule-specific feature. However, fpCCSD produces a slightly wider distribution of errors with a pCCD-optimized basis. On the other hand, XpCCD, XCCD, XfpCCD, and XfpLCCD all show narrower distributions (smaller interquartile ranges) once the pCCD-optimized orbitals are compared. The whiskers for all such graphs elongate, indicating a significant increment in errors in the case of some specific molecules. Examples are SiS, GeS, and GeSe molecules, for which we observe errors >50% for all methods using the pCCD-optimized MOs. Even using the canonical MO basis, the errors for these molecules are already in the range of 30–40% and orbital optimization only worsens them. This is also evident when we compare Figure 2a,b. As we can see, for most molecules, the signed errors reduce in length from the canonical to the pCCD-optimized orbital basis. However, the opposite is true for molecules on the right side of the figure, where we have the diatomics of Group 14 and Group 16 elements, analogous to CO. Notably, for fpLCCSD, the errors reduce in magnitude upon using pCCD-optimized orbitals for these three molecules. Most likely, this is a consequence of incorporating some relaxation effects into the pCCD response density matrix.

**Figure 5:**
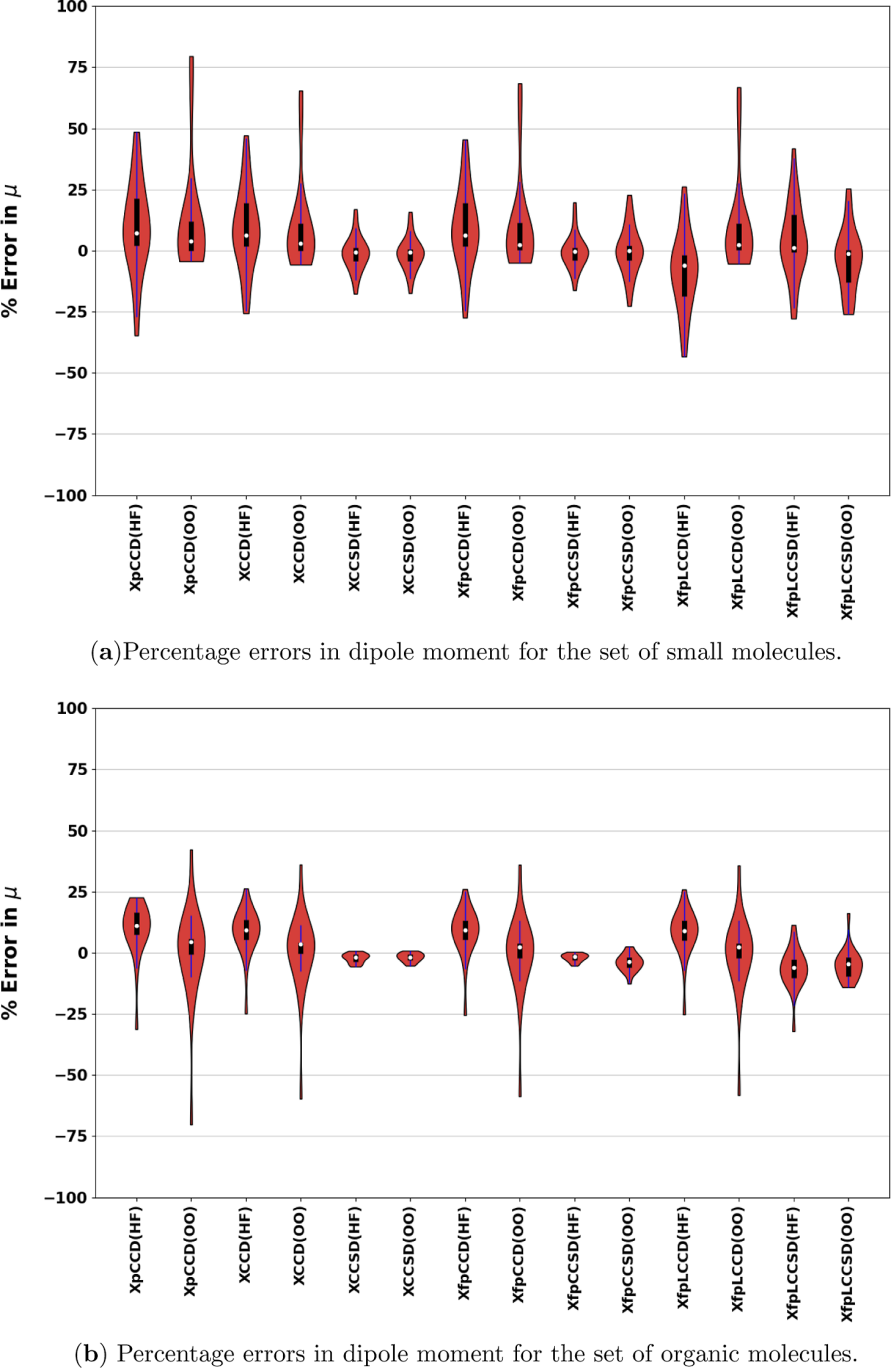
Signed percentage errors w.r.t. response CCSD­(T) dipole moments (μ_Method_ – μ_CCSD(T)_)/(μ_CCSD(T)_) for all methods for (a) small molecules and (b) organic molecules, using the Sadlej-pVTZ basis set. CO and SiSe have been excluded. (HF) and (OO) denote the use of canonical and pCCD-optimized molecular orbitals, respectively.

For organic molecules, on the other hand, all the statistical error parameters for all the methods in Table 3 are lower than the “small” set. pCCD-optimized orbitals further improve the numbers, except for XfpCCSD, for which the performance deteriorates again, and for XCCSD, which remains almost constant. The MAPE for XfpCCSD is remarkably small, ≈2%, using the canonical Mos, exactly similar to that of XCCSD. Figure 3a,b shows the signed errors for the individual organic molecules using canonical and pCCD-optimized MOs, respectively. Their relative errors are depicted in Figure 5b. We observe that the median and interquartile range of all the methods are much closer to the reference compared to the case of the “small” set. They are shifted closer to the reference when pCCD-optimized orbitals are used, except for fpCCSD. However, in the case of optimized orbitals, the minimum percentage error shown by all the methods containing only double excitations (XpCCD, XCCD, XfpCCD, and XfpLCCD) increases significantly. The particular molecule causing this is cyclopentane (19). Its dipole moment length is very small; CCSD­(T) = 0.032 D. Even a tiny difference introduces a significant relative percentage error in such cases. In the case of XfpLCC, going from fpLCCD to fpLCCSD in the case of organic molecules, the RMSE and ME increase in both of the MO bases. This unsatisfactory statistics of XfpLCCSD for organic molecules is largely driven by the high errors shown by it, specifically for nitrobenzene (22) (−1.39 D and −0.46 D in canonical and pCCD-optimized orbital bases). However, this is not much reflected in MAPE as the reference dipole moment of nitrobenzene itself is also high (4.32 D).

#### 4.1.2 Dipole Moment Surface of HF

In the context of prediction of one-electron molecular properties, dipole moment surfaces (DMS) are regular tools to assess a theoretical model’s performance as they reveal the molecule’s rotational–vibrational spectroscopic properties. To that end, in this work, we constructed the DMS of a simple test case of the HF molecule. The molecule is placed along the *z*-axis with H at the origin and F on the positive *z*-axis, and then the bond between the two atoms is stretched along the positive *z*-axis for constructing the DMS. In this analysis, we used pCCD-optimized molecular orbitals. Canonical HF orbital-based pCCD and post-pCCD methods are not size-consistent and also do not produce reliable dipole moment values when the bonds are stretched. Very small strides are taken (0.005 Å), and optimized MOs from the previous geometry are used as the initial guess for the pCCD orbital optimization cycles at the next step. The corresponding potential energy surfaces are shown in Figure S1 in the Supporting Information.

Though reliable at the equilibrium bond distances, CCSD­(T) often fails to estimate dipole moments at nonequilibrium geometries.^87^ Hence, for comparison, we consider here DMS constructed with FCI (subject to SciPy cubic interpolation to get congruent bond distances) at the cc-pVDZ basis set level with canonical molecular orbitals in a previous work by Samanta and Köhn.^86^ Figure 6a shows the DMS constructed with various expectation-value methods at the same basis set level. In this plot, we specifically focus on the XpCCD, XCC, and XfpCC methods. For better visualization, we do not include fpLCC curves here. Around the equilibrium bond distance (0.917 Å), all of the methods are close to each other. When the H—F bond is stretched, initially μ_*z*_ decreases with higher charge density over the F atom, but after a minimum point, the dipole moment starts increasing. The FCI line turns at around 1.27 Å. Other than XCCSD­(OO), all other expectation-value-based curves show a shorter turning point around 1.25 Å. XCCSD­(OO) turns at a longer bond length and shows a flatter curve. It also converges to a lower value of μ_*z*_ than others at the bond-breaking region. This indicates that the XCCSD overestimates the ionic nature of the stretched H—F bond. The XpCCD­(OO) and XfpCCD­(OO) curves almost overlap with each other. The XfpCCSD­(OO), on the other hand, lies almost parallel to the XCCD­(OO) curve. The XfpCCSD­(OO) and XCCD­(OO) curves also show slightly better asymptotic behavior (converging to 0 D at large interatomic distances). Still, the inclusion of single excitations in fpCC does not seem to improve the dipole moments that much at nonequilibrium geometries. This is also evident, as the XCCD­(OO) curve is closer to the FCI line compared to XfpCCSD­(OO).

**Figure 6:**
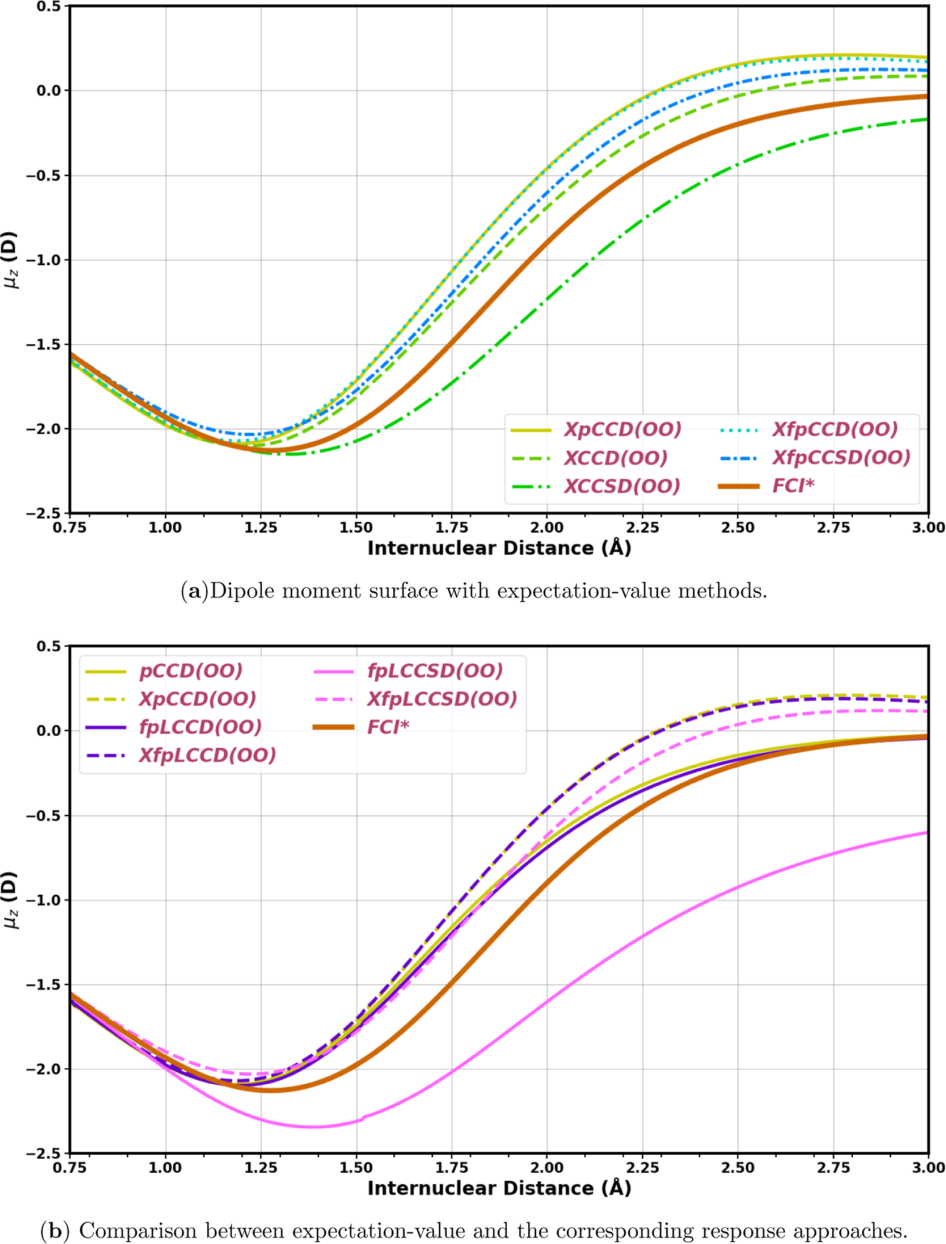
(a) Dipole moment surface of HF in the cc-pVDZ atomic basis set calculated with XpCCD, XCC, and XfpCC methods. (b) Dipole moment surface of HF in the cc-pVDZ atomic basis set calculated with XpCCD, XfpLCC, and their response counterparts. (OO) denotes pCCD-optimized molecular orbitals. In (b), the solid lines represent the response dipole moment surfaces, and the dashed lines show the expectation-value DMS. The FCI* curve is taken from Samanta and Köhn.^86^

In Figure 6b, we show a comparison of the XpCCD and XfpLCC methods with the corresponding response dipole moments from each method. We note that the response pCCD and fpLCCD curves overlap with each other, similar to their respective expectation-value curves. For these two methods, the response curves show lower differences from the reference FCI line compared to the expectation-value curves. Both the pCCD and fpLCCD response DMS also converge with the reference FCI curve at the bond-breaking region. In contrast, the two fpLCCSD curves behave very differently. Specifically, the response fpLCCSD curve shows a large deviation from the FCI curve after 1 Å and the deviation only increases as we go further. The XfpLCCSD curve, on the other hand, remains closer to the FCI curve compared to the other two expectation-value curves. Table 4 provides the nonparallelity errors (maximum deviation—minimum deviation) of all the methods discussed in Figure 6. To summarize, XfpLCCSD shows the overall best performance across the bond-distance regime considered here. Importantly, it shows a marked improvement compared to the response dipole moments using fpLCCSD. Another important aspect is that XfpCCSD and XfpLCCSD perform better than XCCSD at nonequilibrium geometries.

**Table 4:** Non-parallelity Errors of the Methods Compared to the FCI Reference in the cc-pVDZ Basis Set in Figure 6

method	non-parallelity error (D)
pCCD(OO)	0.38
XpCCD(OO)	0.50
XCCD(OO)	0.28
XCCSD(OO)	0.35
XfpCCD(OO)	0.48
XfpCCSD(OO)	0.30
fpLCCD(OO)	0.34
XfpLCCD(OO)	0.49
fpLCCSD(OO)	0.74
XfpLCCSD(OO)	0.29

### 4.2 Quadrupole Moment

In this section, we analyze the quality of the quadrupole moment values obtained using the implemented methods in this work. In Figures 7 and 8, we show the errors in the calculated quadrupole moment components of individual molecules against the response CCSD­(T) values for the “small” set and for organic molecules, respectively. Table 5 details the statistical analysis of the methods for the largest component of the traceless quadrupole moment with respect to the response CCSD­(T) values as the reference. In this case, we excluded ClF from the analysis, which exhibited extremely high relative errors for its quadrupole moment with pCCD, XCCD, XfpCCD, and XfpLCCD in the canonical HF orbital basis. Without any single excitation correction, these methods break down completely for ClF, giving even the order of magnitude wrong for its quadrupole moment (refer to the Supporting Information for the values). Figure 9 shows a comparison of the RMSE of different methods in the two MO bases for the three sets of molecules considered in this work. We focus on the performance of the methods for the “full set” here. The statistical parameters in this case follow a similar pattern to that we saw for the dipole moment. Inclusion of single excitations in the cluster operator vastly improves the overall performance of all the methods. The XfpCCSD quadrupole moments using the canonical MOs come out to be the most reliable, showing slightly lower average errors than XCCSD in the same MO basis. Use of pCCD-optimized orbitals improves the statistical parameters of XCCD, XfpCCD, and XfpLCCSD. However, similar to dipole moment, XfpCCSD follows the reverse trend where the optimized MOs downgrade its performance. In the case of quadrupole moment, we see the same pattern for XfpLCCD as well. Another difference to be observed here is the worsening of the results from XfpLCCD to XfpLCCSD. In the canonical MO basis, the MAE and MAPE between these two methods are almost identical. However, XfpLCCSD shows slightly higher RMSE. This indicates that XfpLCCSD has more outliers. Switching to the pCCD-optimized basis further increases the RMSE.

**Figure 7:**
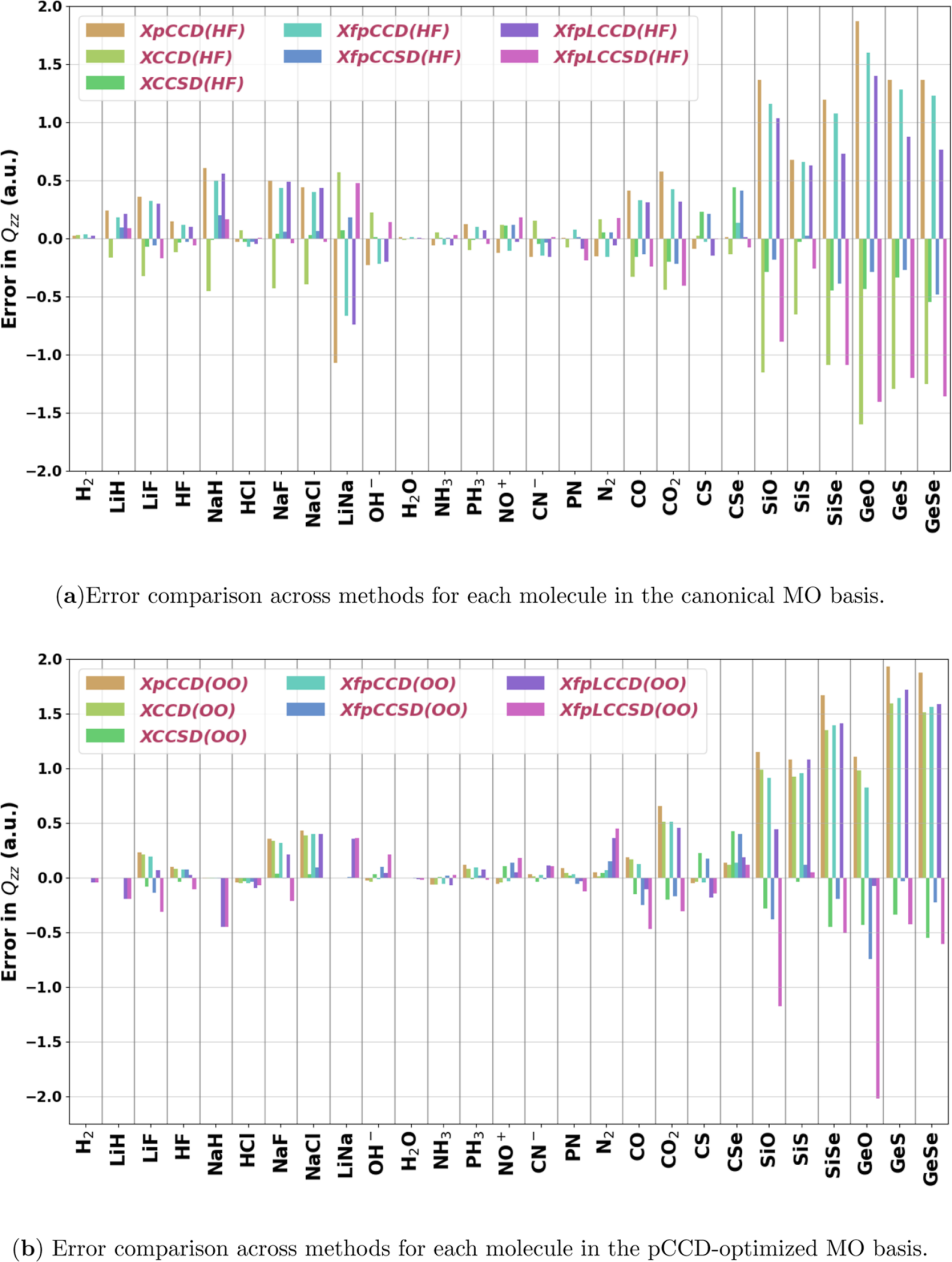
Signed errors with respect to CCSD­(T) in the quadrupole moment component (*Q*_*zz*_^Method^ – *Q*_*zz*_^CCSD(T)^) for all methods using the Sadlej-pVTZ basis set for the set of small molecules investigated in this work. (HF) in (a) and (OO) in (b) denote the use of canonical and pCCD-optimized molecular orbitals, respectively.

**Figure 8:**
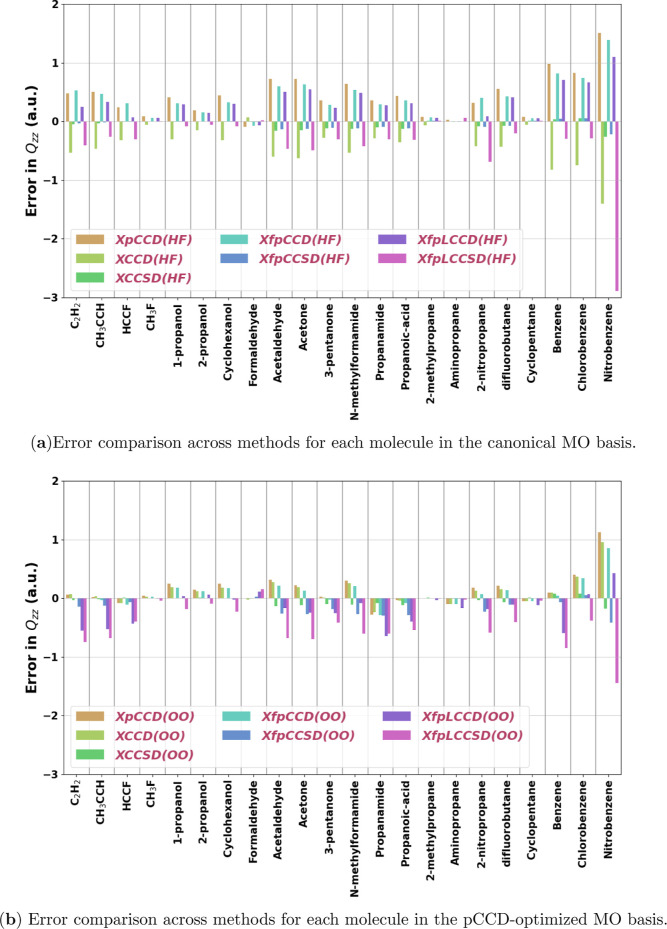
Signed errors with respect to CCSD­(T) in the quadrupole moment component (*Q*_*zz*_^Method^ – *Q*_*zz*_^CCSD(T)^) for all methods using the Sadlej-pVTZ basis set for the set of organic molecules investigated in this work. (HF) in (a) and (OO) in (b) denote the use of canonical and pCCD-optimized molecular orbitals, respectively.

**Table 5:** Statistical Analysis of Various CC- and pCCD-Based Methods with Respect to the Quadrupole Moment (*Q*_*zz*_ in a.u.) Using the Sadlej-pVTZ Basis Set^a^

method	RMSE (a.u.)			ME(a.u.)			MAE (a.u.)			MAPE		
	full set	small	organic	full set	small	organic	full set	small	organic	full set	small	organic
XpCCD(HF)	0.656	0.716	0.573	0.394	0.349	0.450	0.476	0.491	0.458	13.6	15.8	11.0
XpCCD(OO)	0.584	0.737	0.301	0.288	0.407	0.142	0.320	0.424	0.191	10.2	15.3	4.1
XCCD(HF)	0.571	0.616	0.510	–0.354	–0.319	–0.400	0.414	0.423	0.403	12.1	14.3	9.4
XCCD(OO)	0.486	0.613	0.257	0.238	0.338	0.116	0.269	0.355	0.163	8.6	12.6	3.6
XCCSD(HF)	0.170	0.213	0.093	–0.057	–0.061	–0.054	0.103	0.135	0.064	3.8	5.7	1.3
XCCSD(OO)	0.165	0.211	0.075	–0.051	–0.064	–0.036	0.097	0.132	0.054	3.7	5.7	1.2
XfpCCD(HF)	0.571	0.618	0.508	0.353	0.320	0.395	0.416	0.427	0.401	10.2	11.5	8.6
XfpCCD(OO)	0.481	0.613	0.232	0.223	0.336	0.084	0.259	0.351	0.146	8.4	12.7	3.2
XfpCCSD(HF)	0.151	0.190	0.082	–0.035	–0.025	–0.047	0.099	0.133	0.058	3.5	5.4	1.2
XfpCCSD(OO)	0.196	0.209	0.179	–0.073	–0.036	–0.120	0.130	0.131	0.128	4.4	5.8	2.6
XfpLCCD(HF)	0.470	0.512	0.413	0.277	0.250	0.311	0.343	0.363	0.317	9.6	11.2	7.5
XfpLCCD(OO)	0.496	0.606	0.312	0.072	0.273	–0.174	0.309	0.366	0.238	11.1	14.6	6.7
XfpLCCSD(HF)	0.611	0.544	0.684	–0.285	–0.230	–0.353	0.341	0.325	0.362	9.2	11.4	6.5
XfpLCCSD(OO)	0.541	0.525	0.559	–0.309	–0.211	–0.431	0.377	0.324	0.445	11.2	11.9	10.4

a The statistical analysis includes the root-mean-square-error (RMSE): $\sqrt{\left(\sum_{i}^{N}{\left(Q_{zz}^{Method,i}-Q_{zz}^{CCSD\left(T\right),i}\right)}^{2}\right)/N})$ , mean-error (ME): $\sum_{i}^{N}\left(Q_{zz}^{Method,i}-Q_{zz}^{CCSD\left(T\right),i}\right)/N$ , mean-absolute-error (MAE): $\sum_{i}^{N}\left|\left(Q_{zz}^{Method,i}-Q_{zz}^{CCSD\left(T\right),i}\right)\right|/N$ , and mean-absolute-percentage-error (MAPE): ${\overset{N}{\underset{i}{\sum }}[\left|\left(Q_{zz}^{Method,i}-Q_{zz}^{CCSD\left(T\right),i}\right)\right|/(Q_{zz}^{CCSD\left(T\right),i}]}^{*}100/N$ . The molecular sets are described in Section 3.2. (HF) and (OO) denote the use of canonical and pCCD-optimized orbitals, respectively. ClF has been excluded from the analysis.

**Figure 9:**
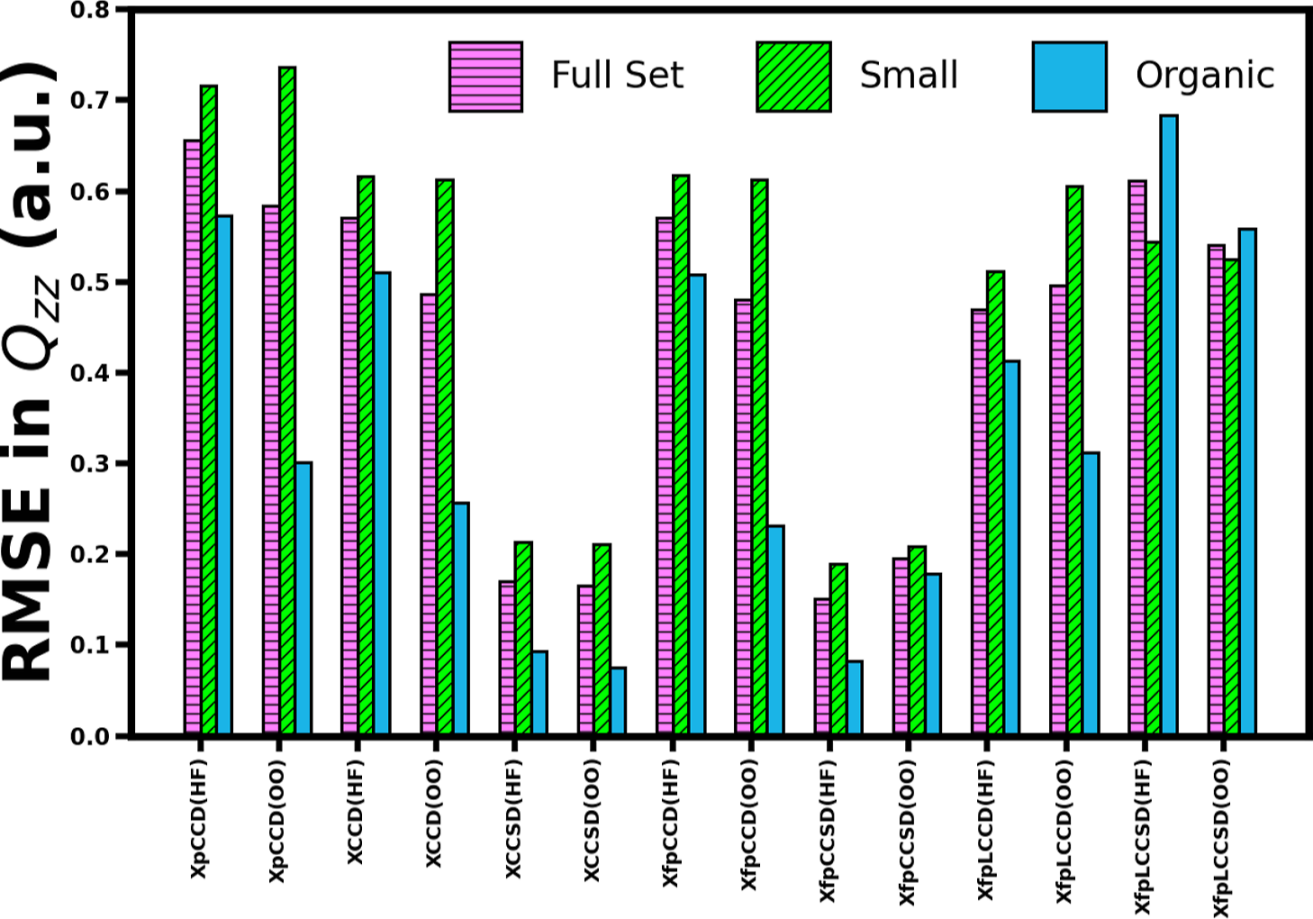
Comparison of root-mean-square-error (RMSE) of quadrupole moment (*Q*_*zz*_ in a.u.) values obtained with various methods. The RMSE is calculated as $\sqrt{\left(\sum_{i}^{N}{\left(Q_{zz}^{Method,i}-Q_{zz}^{CCSD\left(T\right),i}\right)}^{2}\right)/N})$ . The sets of molecules have been described in Section 3.2. (HF) and (OO) denote the use of canonical and pCCD-optimized orbitals, respectively. ClF has been excluded from the analysis.

Moving to the “small” set, estimating the quadrupole moment in the canonical MO basis without the singles excitation seems particularly problematic, as shown by the very high RMSE of XpCCD, XCCD, XfpCCD, and XfpLCCD for this set. In terms of the magnitude of error, again, the diatomics of groups 14 and 16 turned out to be especially challenging, as we can see from Figure 7. For example, for GeO in the canonical MO basis, the errors are 1.87, −1.60, 1.60, and 1.40 a.u. for XpCCD, XCCD, XfpCCD, and XfpLCCD, respectively. The singles correction in fpLCCSD is also inadequate, as errors for XfpLCCSD continue to be high for these molecules. In Figure 10a), we show the distribution of the relative errors in *Q*_*zz*_ for the “small” set. XpCCD, XCCD, and XfpCCD have very similar distributions on the pCCD-optimized MO basis. For these methods, going from canonical to optimized MO basis narrows the relative error distribution and shifts the medians toward the reference. However, the maximum relative errors increase for all three, and the violin plots get elongated. As discussed previously, the molecules responsible for this are SiS, SiSe, GeS, and GeSe. Among the other methods, the distribution of relative error for XfpCCSD in the canonical MO basis is remarkably similar to that of XCCSD. Though pCCD-optimized MOs make the relative error distribution slightly wider for XfpCCSD, the highest error remains under 20% (for CSe). XfpLCCSD behaves differently compared to other methods because its relative error distribution improves to a small extent with pCCD-optimized MOs. The median becomes more negative in this case, although the largest errors are reduced. For example, in the canonical basis for XfpLCCSD, the highest errors are shown by SiSe (58%) and GeSe (%). In the case of pCCD-optimized MOs, the highest errors are given by CO (35%) and SiSe (33%).

**Figure 10:**
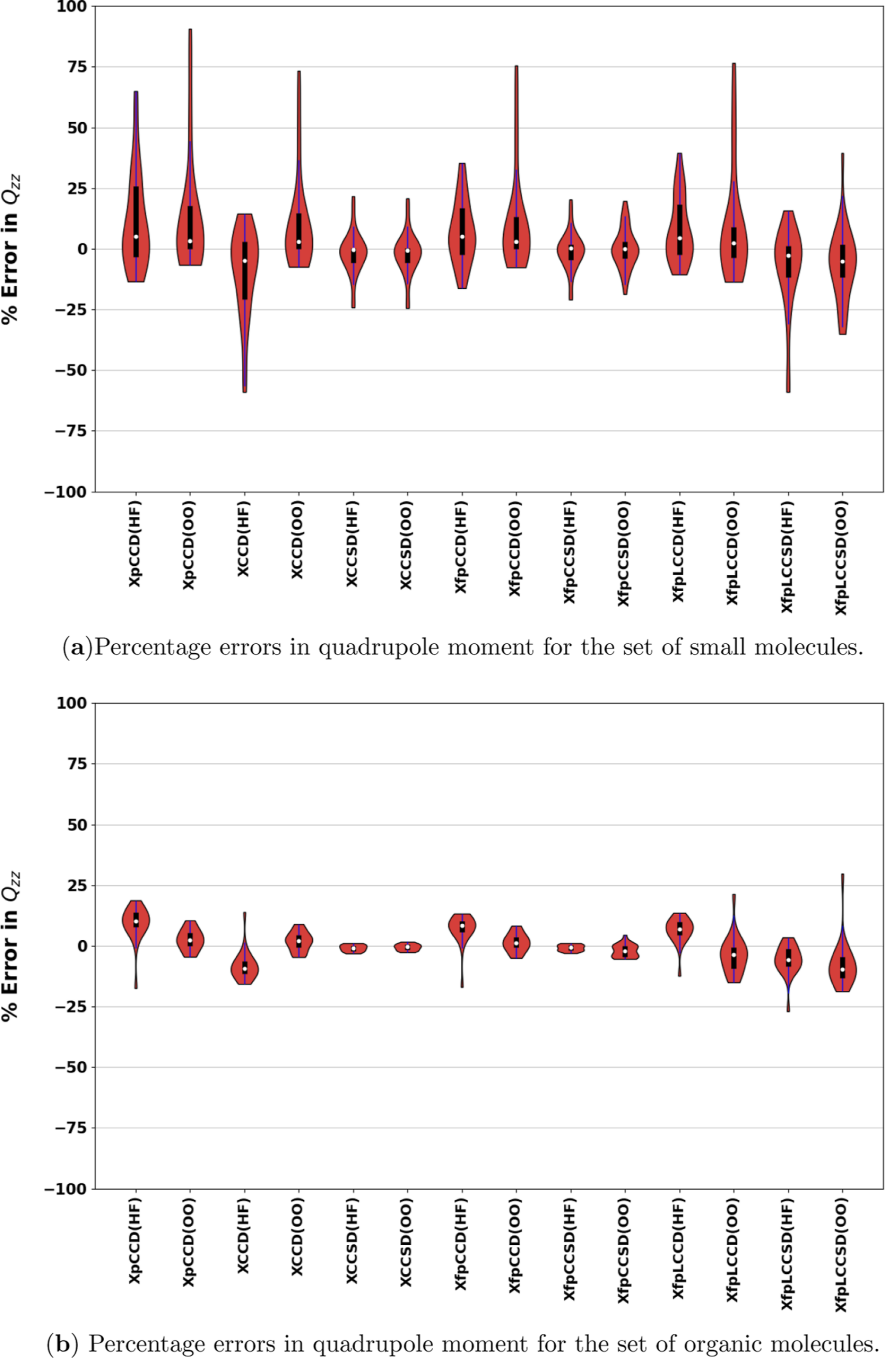
Signed percentage errors with respect to CCSD­(T) reference values in the quadrupole moment component ((*Q*_*zz*_^Method^ – *Q*_*zz*_^CCSD(T)^)/*Q*_*zz*_^CCSD(T)^) for all methods using the Sadlej-pVTZ basis set for the set of (a) small and (b) organic molecules investigated in this work. ClF has been excluded. (HF) and (OO) denote the use of canonical and pCCD-optimized molecular orbitals, respectively. The same range for the *y*-axis has been used in both plots for comparison.

For the case of organic molecules, as shown in Figure 10b, the distributions of relative errors for all of the methods are remarkably small. The effect of optimized orbital follows the same trend as in other cases. XCCSD and XfpCCSD show MAPE as low as 1–2%. The maximum relative error is found for formaldehyde with the doubles-only methods in the canonical MO basis, though these are still under 20%.

## 5 Conclusions

In this work, we implemented and tested the expectation-value-based single-electron property calculation approach for pCCD-based methods. Our approach offers an alternate and computationally cheaper route for computing 1-RDM-based molecular properties to more computationally expensive Λ-equations within the CC response framework. Yet, we implemented the quadrupole moment in the PyBEST software package, which allowed us to compute and benchmark pCCD-based quadrupole moments for the first time.

Our analysis of expectation value pCCD-based dipole and quadrupole moments shows that moving from the canonical HF orbitals to the pCCD-optimized orbital basis reduces errors in all cases except XfpCCSD. That, in turn, highlights that the localized pCCD orbitals are a good choice for computing properties. Moreover, our data suggest that the errors do not emerge from choosing pCCD-optimized localized orbitals but from the method itself—more precisely, from the consecutive treatment of zero and nonzero seniority sectors; splitting the seniority sectors does not improve property values.

Furthermore, our numerical analysis of organic systems shows that XpCCD­(OO) already provides reliable dipole and quadrupole moments; the corresponding errors are similar to those of XCCD, XfpCC, and XfpLCC. Specifically, XfpCCD and XfpLCCD are the best (X)­pCCD-based methods. On the other hand, including single excitations in the fpCC or fpLCC models worsens the performance of pCCD-based models. In contrast to the organic-molecule test set, the performance is reversed for the small-molecule test set. Thus, pCCD-based methods are promising for one-electron properties in organic systems. pCCD orbital optimization is also beneficial for reliably modeling the dipole moment surface of the bond-breaking process of the HF molecule. For this bond-breaking example, however, the response DMS resulted in smaller nonparallelity errors than the corresponding expectation value DMS.

To conclude, we demonstrated that pCCD is a computationally cheap method that provides a good starting point to model one-electron properties in organic systems and systems with a strong electron correlation. The good performance of pCCD-based methods in approximating the 1-RDMs within the expectation value approach makes them a computationally attractive alternative to solving the corresponding CC Λ-equations. This feature is particularly important when calculating orbital entanglement and correlation measures^88,89^ (single orbital entropies and orbital-pair mutual information) from the tailored (or frozen-pair) CC methods.^90^

## Supplementary Material







## Data Availability

The data underlying this study are available in the published article and its Supporting Information. The PyBEST code is available on Zenodo at https://zenodo.org/records/10069179 and on PyPI at https://pypi.org/project/pybest/.

## Notes

Bond distances (in Å) of all the “small” molecules and ions and xyz coordinates (in Å) of all organic molecules considered in this work (PDF)
All the dipole and quadrupole moment values obtained with different expectation-value methods, and response CCSD­(T) and CCSD using the Sadlej pVTZ basis set (XLSX)
Potential energy surface of HF using the cc-pVDZ basis set with all CC variants; deviation of the XpCCD; response pCCD, XCC, XfpCC, and XfpLCC and response fpLCC dipole moment surfaces from the reference FCI surface in the cc-pVDZ basis set; dipole moment surface of HF with the XpCCD, XCC, and XfpCC methods using the Sadlej pVTZ basis set; statistical measurements with respect to CCSD response dipole and quadrupole moment values; dipole moment values at all bond distances calculated during the construction of the dipole moment surface of the HF molecule with all the expectation-value methods discussed here in the cc-pVDZ basis set (PDF)
