# Supplementary material for: Expectation Value-pCCD-Based Methods for Single-Electron Properties
Source: J Phys Chem A. 2025 Jul 16;129(29):6713–32. doi: 10.1021/acs.jpca.5c03859 (PMC12302219; doi:10.1021/acs.jpca.5c03859)
Supplement: Supplementary file 1 [file jp5c03859_si_001.pdf]

Expectation Value-pCCD-based Methods for Single-Electron Properties

Rahul Chakraborty,[1] Somayeh Ahmadkhani,[1,*] Julian Świerczyński,[1] Katharina Boguslawski,[1] and Paweł Tecmer[1,*]

[1] *Institute of Physics, Faculty of Physics, Astronomy and Informatics, Nicolaus Copernicus University in Torun, Grudziadzka 5, 87-100 Torun, Poland*

Corresponding authors: so.ahmadkhani@gmail.com, ptecmer@fizyka.umk.pl

Supplementary Information

S1 Potential energy surface of HF molecule for different methods in cc-pVDZ basis set

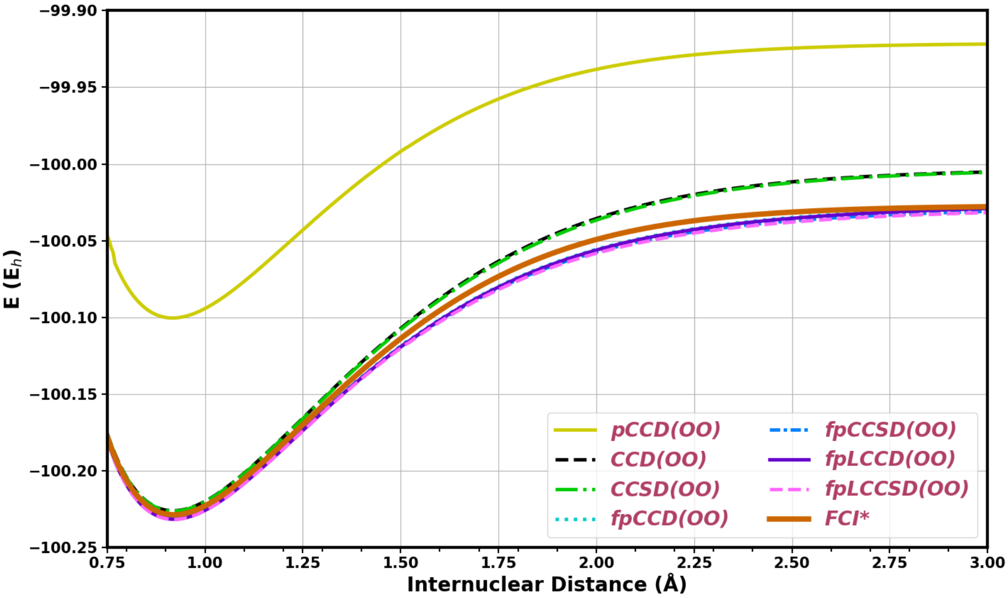

Figure S1: Potential energy surfaces of the HF molecule obtained from different CC flavours investigated in this work using cc-pVDZ atomic basis set. (OO) refers to the use of pCCD-optimized molecular orbitals. The FCI* energies are taken from Samanta et al.[?] .

S2 Deviations in dipole moment surfaces of HF molecule for different methods in cc-pVDZ basis set

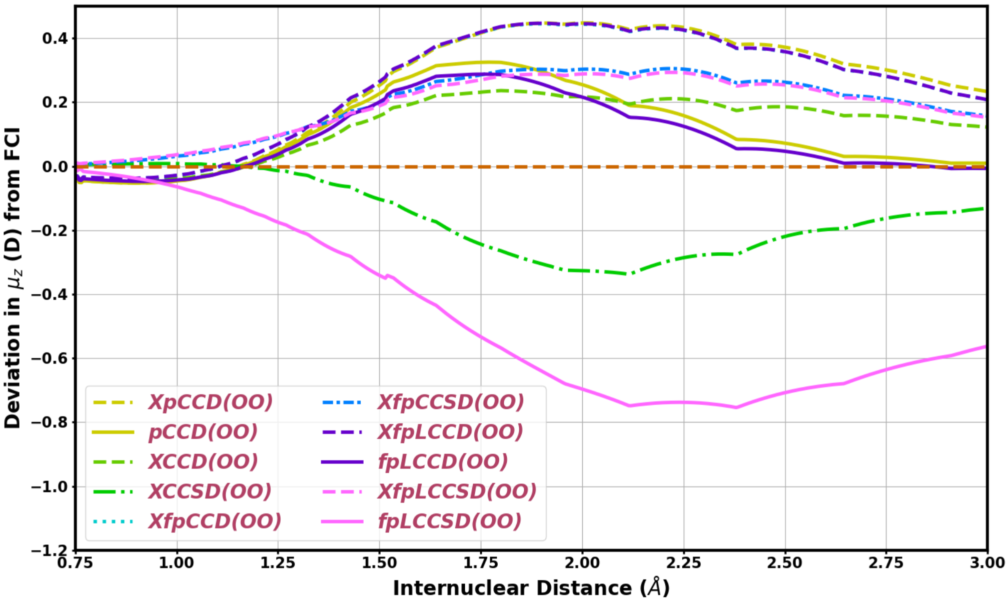

Figure S2: Deviations from the reference FCI/cc-pVDZ curve (($\mu_{\text{Method}} - \mu_{\text{FCI}}$)) for different methods. The horizontal line at 0 D denotes the reference line. (OO) denotes pCCD-optimized molecular orbitals. The FCI* curve is taken from Samanta et al.[?] .

Figure S2 shows the deviations for each method from the reference FCI line calculated as $\mu_{\text{Method}} - \mu_{\text{FCI}}$) at each point. It is to be noted that μ_z is negative in our case. Hence, a positive deviation from FCI indicates underestimation of the dipole moment, and negative deviation meaning vice versa. At shorter distances, XCCSD performs very well and lies almost on the horizontal line at error = 0 D. However, from 1.25 Å onwards it starts overestimating the dipole moment, showing higher magnitude of μ_z and negative deviation. XpCCD, XCCD, and XfpCCD start with showing negative deviation and cross the reference FCI line at around 1.15 Å. On the other hand, XfpCCSD underestimates the dipole moment values throughout the bond distances. Response fpLCCD DMS shows better behaviour than its expectation-value counterpart, whereas the opposite is true for fpLCCSD.

S3 Dipole moment surface of HF in Sadlej pVTZ basis set

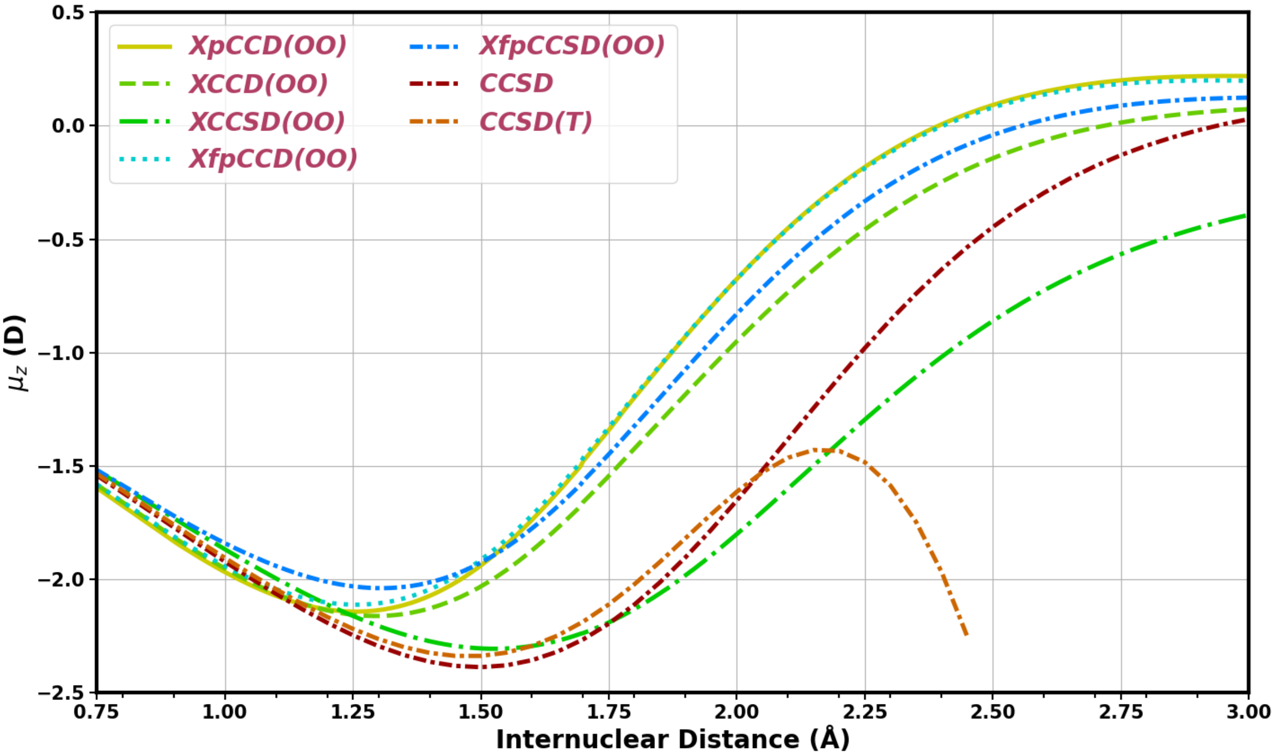

Figure S3: Dipole moment surface of HF in Sadlej-pVTZ atomic basis set calculated with different methods. (OO) denotes pCCD-optimized orbitals. Orbital relaxed CCSD and CCSD(T) response dipole moment curves calculated with canonical HF molecular orbitals are shown for comparison. The apparent breakdown of CCSD(T) after 2.15 Å is to be noted.

S4 Statistical measurements for dipole moment against CCSD as reference

Table S1: Statistical analysis of performances of various methods with respect to the dipole moment (μ in D) values obtained with them using Sadlej-pVTZ basis set. The three performance measures analyzed here are- root-mean-square-error (RMSE): $\sqrt{(\sum_i^N(\mu_{\mathrm{Method,i}} - \mu_{\mathrm{CCSD,i}})^2)/N)}$, mean-error (ME): $\sum_i^N(\mu_{\mathrm{Method,i}} - \mu_{\mathrm{CCSD,i}}))/N$, mean-absolute-error (MAE): $\sum_i^N |(\mu_{\mathrm{Method,i}} - \mu_{\mathrm{CCSD,i}})|)/N$, and mean-absolute-percentage-error (MAPE): $\sum_i^N[|(\mu_{\mathrm{Method,i}} - \mu_{\mathrm{CCSD,i}})|/(\mu_{\mathrm{CCSD,i}}] * 100/N$. The three sets of molecules have been described in section *Computational Details* in the main article. (HF) and (OO) denote the use of canonical and pCCD-optimized orbitals, respectively. CO and SiSe have been excluded from the analysis.

Method	RMSE (D)			ME (D)			MAE (D)			MAPE		
	Full set	Small	Organic	Full set	Small	Organic	Full set	Small	Organic	Full set	Small	Organic
XpCCD(HF)	0.288	0.298	0.276	0.179	0.142	0.222	0.236	0.248	0.224	12.0	12.7	11.1
XpCCD(OO)	0.254	0.332	0.107	0.117	0.167	0.059	0.137	0.181	0.087	10.0	10.2	9.9
XCCD(HF)	0.253	0.265	0.237	0.154	0.126	0.186	0.205	0.221	0.188	10.3	11.2	9.3
XCCD(OO)	0.197	0.257	0.086	0.088	0.128	0.043	0.111	0.148	0.068	8.2	8.3	8.1
XCCSD(HF)	0.180	0.225	0.108	-0.080	-0.074	-0.087	0.124	0.154	0.088	6.1	7.6	4.3
XCCSD(OO)	0.173	0.219	0.095	-0.076	-0.074	-0.077	0.116	0.149	0.079	5.7	7.3	4.0
XfpCCD(HF)	0.247	0.256	0.236	0.155	0.128	0.186	0.201	0.214	0.187	10.2	11.0	9.3
XfpCCD(OO)	0.200	0.264	0.080	0.077	0.126	0.021	0.110	0.151	0.063	8.4	8.7	8.0
XfpCCSD(HF)	0.174	0.219	0.100	-0.066	-0.053	-0.081	0.121	0.154	0.083	5.8	7.3	4.0
XfpCCSD(OO)	0.229	0.270	0.171	-0.097	-0.067	-0.132	0.157	0.179	0.132	7.2	8.3	6.0
XfpLCCD(HF)	0.235	0.243	0.225	0.147	-0.121	0.177	0.192	0.204	0.178	9.8	10.4	9.0
XfpLCCD(OO)	0.194	0.255	0.078	0.073	0.120	0.018	0.108	0.149	0.061	8.2	8.6	7.9
XfpLCCSD(HF)	0.401	0.402	0.401	-0.185	0.139	-0.238	0.256	0.270	0.241	11.9	13.2	10.3
XfpLCCSD(OO)	0.322	0.384	0.233	-0.155	-0.137	-0.176	0.219	0.256	0.178	10.4	12.0	8.6

S5 Statistical measurements for quadrupole moment against CCSD as reference

Table S2: Statistical analysis of performances of various methods with respect to the quadrupole moment (Q_{zz} in a.u.) values obtained with them using Sadlej-pVTZ basis set. The three performance measures analyzed here are- root-mean-square-error (RMSE): $\sqrt{(\sum_i^N (Q_{zz}^{\mathrm{Method,i}} - Q_{zz}^{\mathrm{CCSD,i}})^2)/N)}$, mean-error (ME): $\sum_i^N (Q_{zz}^{\mathrm{Method,i}} - Q_{zz}^{\mathrm{CCSD,i}})/N$, mean-absolute-error (MAE): $\sum_i^N |(Q_{zz}^{\mathrm{Method,i}} - Q_{zz}^{\mathrm{CCSD,i}})|/N$, and mean-absolute-percentage-error (MAPE): $\sum_i^N [|(Q_{zz}^{\mathrm{Method,i}} - Q_{zz}^{\mathrm{CCSD,i}})|/(Q_{zz}^{\mathrm{CCSD,i}}] * 100/N$. The three sets of molecules have been described in section *Computational Details* in the main article. (HF) and (OO) denote the use of canonical and pCCD-optimized orbitals, respectively. ClF has been excluded from the analysis.

Method	RMSE (D)			ME (D)			MAE (D)			MAPE		
	Full set	Small	Organic	Full set	Small	Organic	Full set	Small	Organic	Full set	Small	Organic
XpCCD(HF)	0.552	0.599	0.487	0.325	0.273	0.388	0.412	0.426	0.395	11.5	13.3	9.4
XpCCD(OO)	0.483	0.619	0.224	0.218	0.331	0.080	0.256	0.350	0.141	8.1	12.2	3.2
XCCD(HF)	0.463	0.492	0.425	-0.284	-0.243	-0.335	0.343	0.345	0.340	9.8	11.3	7.9
XCCD(OO)	0.385	0.493	0.180	0.169	0.262	0.054	0.206	0.281	0.113	6.5	9.7	2.5
XCCSD(HF)	0.262	0.318	0.169	-0.127	-0.136	-0.115	0.160	0.195	0.117	4.9	7.0	2.2
XCCSD(OO)	0.255	0.317	0.145	-0.121	-0.140	-0.098	0.150	0.191	0.100	4.7	6.9	1.9
XfpCCD(HF)	0.464	0.496	0.422	0.283	0.244	0.333	0.344	0.349	0.338	8.5	9.6	7.3
XfpCCD(OO)	0.385	0.497	0.164	0.154	0.261	0.022	0.204	0.280	0.112	6.6	9.8	2.7
XfpCCSD(HF)	0.237	0.286	0.157	-0.104	-0.101	-0.108	0.152	0.186	0.111	4.5	6.5	2.1
XfpCCSD(OO)	0.283	0.305	0.252	-0.143	-0.111	-0.182	0.183	0.180	0.185	5.4	6.7	3.8
XfpLCCD(HF)	0.370	0.400	0.329	0.208	0.174	0.250	0.279	0.299	0.254	7.6	8.9	6.0
XfpLCCD(OO)	0.448	0.521	0.339	0.003	0.197	-0.236	0.298	0.327	0.262	10.2	12.4	7.4
XfpLCCSD(HF)	0.713	0.668	0.765	-0.355	-0.306	-0.415	0.415	0.406	0.425	10.8	13.3	7.7
XfpLCCSD(OO)	0.639	0.638	0.641	-0.379	-0.286	-0.492	0.443	0.390	0.508	12.6	13.3	11.7

S6 Dipole moment values of HF at different bond lengths

Table S3: Dipole moments (in D) of the HF molecule with the cc-pVDZ basis set calculated with the different expectation-value methods. OO denotes pCCD-optimized molecular orbitals.

r(Å)	XpCCD(OO)	XCCD(OO)	XCCSD(OO)	XfpCCD(OO)	XfpCCSD(OO)	XfpLCCD(OO)	XfpLCCSD(OO)
0.750	-1.599155	-1.589988	-1.588570	-1.55081	-1.588235	-1.593182	-1.571930
0.755	-1.607283	-1.598066	-1.596569	-1.558356	-1.596313	-1.601414	-1.580468
0.760	-1.624447	-1.614320	-1.613515	-1.568842	-1.613326	-1.617612	-1.583658
0.765	-1.633130	-1.622888	-1.622034	-1.576523	-1.621746	-1.626179	-1.592128
0.770	-1.635039	-1.625248	-1.623971	-1.583046	-1.623724	-1.629050	-1.606332
0.775	-1.643481	-1.633615	-1.632268	-1.590670	-1.631982	-1.637450	-1.614871
0.780	-1.651885	-1.641943	-1.640525	-1.598295	-1.640245	-1.645857	-1.623424
0.785	-1.660284	-1.650281	-1.648784	-1.605897	-1.648487	-1.654246	-1.631996
0.790	-1.668621	-1.658550	-1.656975	-1.613505	-1.656705	-1.662613	-1.640580
0.795	-1.676984	-1.666862	-1.665200	-1.621085	-1.664915	-1.670974	-1.649180
0.800	-1.685291	-1.675115	-1.673366	-1.628664	-1.673091	-1.679305	-1.657789
0.805	-1.693678	-1.683456	-1.681614	-1.636226	-1.681286	-1.687657	-1.666408
0.810	-1.702018	-1.691742	-1.689810	-1.643792	-1.689467	-1.695997	-1.675032
0.815	-1.710296	-1.699982	-1.697950	-1.651323	-1.697593	-1.704285	-1.683669
0.820	-1.718615	-1.708255	-1.706125	-1.658853	-1.705730	-1.712586	-1.692308
0.825	-1.726854	-1.716462	-1.714226	-1.666351	-1.713814	-1.720838	-1.700955
0.830	-1.735080	-1.724645	-1.722306	-1.673849	-1.721882	-1.729075	-1.709605
0.835	-1.743135	-1.732699	-1.730236	-1.681261	-1.729851	-1.737219	-1.718264
0.840	-1.751397	-1.740930	-1.738355	-1.688712	-1.737896	-1.745439	-1.726913
0.845	-1.759415	-1.748935	-1.746238	-1.696106	-1.745817	-1.753539	-1.735572
0.850	-1.767461	-1.756976	-1.754152	-1.703471	-1.753723	-1.761627	-1.744225
0.855	-1.775472	-1.764980	-1.762026	-1.710815	-1.761600	-1.769689	-1.752876
0.860	-1.783494	-1.772997	-1.769911	-1.718132	-1.769454	-1.777731	-1.761520
0.865	-1.791366	-1.780881	-1.777654	-1.725397	-1.777212	-1.785680	-1.770165
0.870	-1.799252	-1.788776	-1.785406	-1.732638	-1.784952	-1.793614	-1.778800
0.875	-1.807183	-1.796716	-1.793201	-1.739854	-1.792679	-1.801538	-1.787424
0.880	-1.814928	-1.804478	-1.800812	-1.747021	-1.800275	-1.809335	-1.796047
0.885	-1.822610	-1.812195	-1.808367	-1.754127	-1.807854	-1.817118	-1.804656
0.890	-1.830262	-1.819879	-1.815889	-1.761205	-1.815389	-1.824859	-1.813251
0.895	-1.837862	-1.827514	-1.823357	-1.768239	-1.822852	-1.832532	-1.821835
0.900	-1.845378	-1.835076	-1.830745	-1.775222	-1.830239	-1.840132	-1.830408
0.905	-1.852871	-1.842615	-1.838108	-1.782164	-1.837584	-1.847694	-1.838961
0.910	-1.860232	-1.850035	-1.845343	-1.789042	-1.844830	-1.855161	-1.847501
0.915	-1.867791	-1.857635	-1.852764	-1.795913	-1.852117	-1.862671	-1.856018
0.920	-1.874942	-1.864874	-1.859799	-1.802660	-1.859192	-1.869973	-1.864523
0.925	-1.882025	-1.872033	-1.866757	-1.809374	-1.866198	-1.877208	-1.873005
0.930	-1.889113	-1.879208	-1.873724	-1.816028	-1.873156	-1.884400	-1.881461
0.935	-1.896124	-1.886314	-1.880614	-1.822623	-1.880035	-1.891515	-1.889892
0.940	-1.903055	-1.893350	-1.887427	-1.829152	-1.886832	-1.898552	-1.898296
0.945	-1.909941	-1.900346	-1.894195	-1.835658	-1.893584	-1.905547	-1.906708
0.950	-1.916659	-1.907192	-1.900801	-1.842045	-1.900190	-1.912399	-1.915058
0.955	-1.923290	-1.913959	-1.907322	-1.848363	-1.906710	-1.919167	-1.923377
0.960	-1.929876	-1.920687	-1.913797	-1.854618	-1.913152	-1.925861	-1.931662
0.965	-1.936307	-1.927276	-1.920123	-1.860787	-1.919476	-1.932441	-1.939915
0.970	-1.942648	-1.933784	-1.926361	-1.866881	-1.925707	-1.938930	-1.948133

Continued on next page

Table S3 – Continued from previous page

r(Å)	pCCD(OO)	CCD(OO)	CCSD(OO)	fpCCD(OO)	fpCCSD(OO)	fpLCCD(OO)	fpLCCSD(OO)
0.975	-1.948899	-1.940212	-1.932511	-1.872900	-1.931843	-1.945328	-1.956312
0.980	-1.955004	-1.946511	-1.938521	-1.878828	-1.937858	-1.951607	-1.964456
0.985	-1.961032	-1.952739	-1.944454	-1.884681	-1.943778	-1.957795	-1.972559
0.990	-1.966924	-1.958845	-1.950254	-1.890444	-1.949581	-1.963869	-1.980620
0.995	-1.972728	-1.964874	-1.955969	-1.896125	-1.955283	-1.969845	-1.988638
1.000	-1.978447	-1.970827	-1.961601	-1.901721	-1.960883	-1.975723	-1.996610
1.005	-1.983972	-1.976604	-1.967044	-1.907216	-1.966339	-1.981460	-2.004540
1.010	-1.989419	-1.982312	-1.972410	-1.912623	-1.971693	-1.987097	-2.012420
1.015	-1.994744	-1.987912	-1.977658	-1.917936	-1.976927	-1.992619	-2.020250
1.020	-1.999933	-1.993389	-1.982773	-1.923151	-1.982033	-1.998015	-2.028030
1.025	-2.004999	-1.998755	-1.987768	-1.928268	-1.987016	-2.003292	-2.035757
1.030	-2.010172	-2.004222	-1.992862	-1.933323	-1.992038	-2.008612	-2.043420
1.035	-2.014747	-2.009145	-1.997384	-1.938200	-1.996606	-2.013478	-2.051048
1.040	-2.019417	-2.014157	-2.001993	-1.943011	-2.001204	-2.018379	-2.058609
1.045	-2.023951	-2.019047	-2.006469	-1.947715	-2.005668	-2.023149	-2.066110
1.050	-2.028343	-2.023808	-2.010806	-1.952313	-2.009998	-2.027788	-2.073551
1.055	-2.032621	-2.028469	-2.015033	-1.956803	-2.014205	-2.032307	-2.080928
1.060	-2.037024	-2.033244	-2.019374	-1.961230	-2.018466	-2.036886	-2.088229
1.065	-2.040707	-2.037367	-2.023028	-1.965445	-2.022178	-2.040912	-2.095489
1.070	-2.044508	-2.041599	-2.026790	-1.969591	-2.025936	-2.044990	-2.102671
1.075	-2.048187	-2.045721	-2.030432	-1.973623	-2.029563	-2.048941	-2.109781
1.080	-2.051822	-2.049809	-2.034031	-1.977553	-2.033123	-2.052829	-2.116816
1.085	-2.055094	-2.053562	-2.037277	-1.981333	-2.036378	-2.056413	-2.123786
1.090	-2.058544	-2.057480	-2.040689	-1.985047	-2.039728	-2.060098	-2.130670
1.095	-2.061386	-2.060851	-2.043522	-1.988558	-2.042600	-2.063302	-2.137492
1.100	-2.064291	-2.064280	-2.046409	-1.991984	-2.045482	-2.066521	-2.144228
1.105	-2.067050	-2.067577	-2.049154	-1.995285	-2.048212	-2.069592	-2.150883
1.110	-2.069636	-2.070719	-2.051730	-1.998458	-2.050784	-2.072506	-2.157456
1.115	-2.072125	-2.073778	-2.054212	-2.001509	-2.053230	-2.075299	-2.163940
1.120	-2.074375	-2.076617	-2.056459	-2.004422	-2.055474	-2.077891	-2.170343
1.125	-2.076542	-2.079388	-2.058628	-2.007210	-2.057618	-2.080385	-2.176654
1.130	-2.078524	-2.081989	-2.060613	-2.009868	-2.059583	-2.082703	-2.182872
1.135	-2.080178	-2.084291	-2.062281	-2.012366	-2.061271	-2.084744	-2.189007
1.140	-2.081986	-2.086741	-2.064094	-2.014777	-2.063015	-2.086848	-2.195032
1.145	-2.083235	-2.088681	-2.065370	-2.016991	-2.064336	-2.088525	-2.200977
1.150	-2.084551	-2.090690	-2.066709	-2.019099	-2.065648	-2.090197	-2.206812
1.155	-2.085606	-2.092458	-2.067792	-2.021061	-2.066728	-2.091637	-2.212547
1.160	-2.086536	-2.094116	-2.068752	-2.022887	-2.067674	-2.092946	-2.218181
1.165	-2.087299	-2.095623	-2.069549	-2.024576	-2.068471	-2.094105	-2.223707
1.170	-2.087894	-2.096982	-2.070184	-2.026122	-2.069094	-2.095092	-2.229122
1.175	-2.088376	-2.098245	-2.070709	-2.027528	-2.069581	-2.095945	-2.234424
1.180	-2.088583	-2.099250	-2.070962	-2.028783	-2.069826	-2.096554	-2.239616
1.185	-2.088618	-2.100100	-2.071047	-2.029896	-2.069933	-2.097025	-2.244694
1.190	-2.088580	-2.100893	-2.071063	-2.030870	-2.069894	-2.097353	-2.249646
1.195	-2.088335	-2.101498	-2.070877	-2.031696	-2.069687	-2.097513	-2.254486
1.200	-2.087734	-2.101770	-2.070341	-2.032353	-2.069186	-2.097374	-2.259208
1.205	-2.087105	-2.102025	-2.069777	-2.032879	-2.068618	-2.097173	-2.263806
1.210	-2.086301	-2.102123	-2.069042	-2.033253	-2.067859	-2.096781	-2.268274
1.215	-2.085334	-2.102071	-2.068145	-2.033480	-2.066935	-2.096223	-2.272618
1.220	-2.084140	-2.101816	-2.067030	-2.033546	-2.065817	-2.095473	-2.276839

Continued on next page

Table S3 – Continued from previous page

r(Å)	pCCD(OO)	CCD(OO)	CCSD(OO)	fpCCD(OO)	fpCCSD(OO)	fpLCCD(OO)	fpLCCSD(OO)
1.225	-2.082862	-2.101484	-2.065827	-2.033473	-2.064584	-2.094611	-2.280929
1.230	-2.081362	-2.100948	-2.064404	-2.033240	-2.063135	-2.093532	-2.284890
1.235	-2.079435	-2.100014	-2.062565	-2.032819	-2.061351	-2.092117	-2.288734
1.240	-2.077522	-2.099102	-2.060738	-2.032262	-2.059504	-2.090646	-2.292439
1.245	-2.075413	-2.098008	-2.058714	-2.031545	-2.057470	-2.088991	-2.296016
1.250	-2.073104	-2.096732	-2.056495	-2.030665	-2.055236	-2.087138	-2.299460
1.255	-2.070597	-2.095275	-2.054080	-2.029620	-2.052816	-2.085104	-2.302776
1.260	-2.067885	-2.093630	-2.051461	-2.028407	-2.050186	-2.082863	-2.305956
1.265	-2.064985	-2.091813	-2.048656	-2.027027	-2.047381	-2.080452	-2.309007
1.270	-2.061865	-2.089794	-2.045635	-2.025476	-2.044354	-2.077824	-2.311919
1.275	-2.058745	-2.087775	-2.042604	-2.023787	-2.041259	-2.075142	-2.314703
1.280	-2.055297	-2.085448	-2.039249	-2.021912	-2.037891	-2.072186	-2.317358
1.285	-2.051568	-2.082870	-2.035623	-2.019841	-2.034244	-2.068957	-2.319861
1.290	-2.047303	-2.079802	-2.031478	-2.017529	-2.030172	-2.065295	-2.322198
1.295	-2.043183	-2.076860	-2.027459	-2.015110	-2.026138	-2.061694	-2.324431
1.300	-2.038795	-2.073672	-2.023175	-2.012497	-2.021835	-2.057830	-2.326513
1.305	-2.034362	-2.070451	-2.018845	-2.009729	-2.017460	-2.053908	-2.328480
1.310	-2.029265	-2.066600	-2.013863	-2.006701	-2.012516	-2.049410	-2.330256
1.315	-2.024180	-2.062768	-2.008887	-2.003530	-2.007531	-2.044891	-2.331938
1.320	-2.018868	-2.058727	-2.003686	-2.000174	-2.002327	-2.040165	-2.333502
1.325	-2.013336	-2.054487	-1.998268	-1.996630	-1.996886	-2.035214	-2.334941
1.330	-2.007559	-2.050016	-1.992604	-1.992908	-1.991228	-2.030058	-2.336276
1.335	-2.001557	-2.045345	-1.986722	-1.989000	-1.985333	-2.024679	-2.337508
1.340	-1.995363	-2.040500	-1.980651	-1.984923	-1.979251	-2.019126	-2.338633
1.345	-1.988983	-2.035489	-1.974398	-1.980678	-1.972984	-2.013403	-2.339656
1.350	-1.982398	-2.030293	-1.967943	-1.976261	-1.966515	-2.007491	-2.340575
1.355	-1.975783	-2.025072	-1.961453	-1.971722	-1.960011	-2.001560	-2.341404
1.360	-1.968660	-2.019376	-1.954467	-1.966938	-1.953056	-1.995180	-2.342097
1.365	-1.961704	-2.013856	-1.947647	-1.962068	-1.946192	-1.988917	-2.342704
1.370	-1.954425	-2.008033	-1.940508	-1.957006	-1.939051	-1.982384	-2.343185
1.375	-1.946959	-2.002040	-1.933185	-1.951777	-1.931728	-1.975681	-2.343547
1.380	-1.939600	-1.996159	-1.925963	-1.946439	-1.924419	-1.969009	-2.343784
1.385	-1.931986	-1.990039	-1.918489	-1.940931	-1.916915	-1.962152	-2.343902
1.390	-1.923716	-1.983302	-1.910377	-1.935166	-1.908891	-1.954781	-2.343897
1.395	-1.915852	-1.976952	-1.902650	-1.929363	-1.901132	-1.947693	-2.343759
1.400	-1.907727	-1.970365	-1.894669	-1.923380	-1.893141	-1.940387	-2.343491
1.405	-1.899576	-1.963762	-1.886663	-1.917263	-1.885083	-1.933028	-2.343086
1.410	-1.891175	-1.956922	-1.878408	-1.910977	-1.876818	-1.925472	-2.342552
1.415	-1.882426	-1.949756	-1.869810	-1.904530	-1.868311	-1.917677	-2.341935
1.420	-1.873844	-1.942753	-1.861369	-1.897963	-1.859834	-1.909935	-2.341132
1.425	-1.864928	-1.935441	-1.852605	-1.891201	-1.851081	-1.901927	-2.340198
1.430	-1.856115	-1.928220	-1.843928	-1.884350	-1.842375	-1.893982	-2.339124
1.435	-1.847010	-1.920737	-1.834972	-1.877310	-1.833400	-1.885779	-2.337912
1.440	-1.837778	-1.913133	-1.825886	-1.870126	-1.824308	-1.877473	-2.336572
1.445	-1.828469	-1.905456	-1.816720	-1.862812	-1.815137	-1.869101	-2.335101
1.450	-1.818982	-1.897617	-1.807379	-1.855336	-1.805771	-1.860548	-2.333489
1.455	-1.809414	-1.889695	-1.797950	-1.847737	-1.796336	-1.851940	-2.331751
1.460	-1.799484	-1.881449	-1.788177	-1.839931	-1.786601	-1.843042	-2.329881
1.465	-1.789641	-1.873277	-1.778478	-1.832038	-1.776868	-1.834167	-2.327872
1.470	-1.779658	-1.864976	-1.768640	-1.824002	-1.767001	-1.825170	-2.325727

Continued on next page

Table S3 – Continued from previous page

r(Å)	pCCD(OO)	CCD(OO)	CCSD(OO)	fpCCD(OO)	fpCCSD(OO)	fpLCCD(OO)	fpLCCSD(OO)
1.475	-1.769519	-1.856528	-1.758646	-1.815819	-1.756990	-1.816043	-2.323455
1.480	-1.759001	-1.847729	-1.748286	-1.807439	-1.746713	-1.806663	-2.321079
1.485	-1.748725	-1.839153	-1.738149	-1.799004	-1.736520	-1.797383	-2.318538
1.490	-1.738202	-1.830350	-1.727777	-1.790405	-1.726112	-1.787909	-2.315874
1.495	-1.727686	-1.821540	-1.717396	-1.781708	-1.715686	-1.778429	-2.313083
1.500	-1.716716	-1.812314	-1.706583	-1.772808	-1.704953	-1.768651	-2.310172
1.505	-1.705796	-1.803127	-1.695807	-1.763809	-1.694192	-1.758864	-2.307137
1.510	-1.694820	-1.793883	-1.684970	-1.754695	-1.683360	-1.749021	-2.303973
1.515	-1.683615	-1.784446	-1.673926	-1.745414	-1.672270	-1.738945	-2.300669
1.520	-1.663418	-1.770725	-1.656488	-1.729009	-1.655081	-1.720319	-2.285734
1.525	-1.652692	-1.761732	-1.645901	-1.719637	-1.644185	-1.710484	-2.282135
1.530	-1.641006	-1.751803	-1.634362	-1.709975	-1.632806	-1.700150	-2.278442
1.535	-1.629668	-1.742211	-1.623159	-1.700271	-1.621493	-1.689926	-2.274625
1.540	-1.617777	-1.732103	-1.611422	-1.690346	-1.609804	-1.679328	-2.270704
1.545	-1.605962	-1.722060	-1.599753	-1.680350	-1.598165	-1.668804	-2.266674
1.550	-1.594195	-1.712049	-1.588116	-1.670270	-1.586510	-1.658280	-2.262539
1.555	-1.582185	-1.701828	-1.576261	-1.660036	-1.574592	-1.647521	-2.258273
1.560	-1.570147	-1.691550	-1.564353	-1.649723	-1.562694	-1.636791	-2.253916
1.565	-1.557810	-1.681009	-1.552168	-1.639234	-1.550505	-1.625786	-2.249450
1.570	-1.545849	-1.670812	-1.540339	-1.628755	-1.538556	-1.615056	-2.244867
1.575	-1.533390	-1.660122	-1.528010	-1.618089	-1.526321	-1.604030	-2.240201
1.580	-1.520935	-1.649449	-1.515696	-1.607330	-1.513977	-1.592931	-2.235416
1.585	-1.507981	-1.638326	-1.502914	-1.596348	-1.501190	-1.581409	-2.230548
1.590	-1.495601	-1.627682	-1.490639	-1.585463	-1.488944	-1.570434	-2.225579
1.595	-1.482455	-1.616351	-1.477650	-1.574283	-1.476020	-1.558798	-2.220532
1.600	-1.469616	-1.605328	-1.464976	-1.563099	-1.463238	-1.547344	-2.215367
1.605	-1.456701	-1.594182	-1.452191	-1.551849	-1.450452	-1.535898	-2.210069
1.610	-1.443741	-1.582995	-1.439368	-1.540515	-1.437626	-1.524424	-2.204696
1.615	-1.430654	-1.571663	-1.426406	-1.529101	-1.424786	-1.512943	-2.199243
1.620	-1.417513	-1.560289	-1.413399	-1.517586	-1.411813	-1.501351	-2.193687
1.625	-1.404440	-1.548984	-1.400469	-1.506016	-1.398755	-1.489709	-2.188003
1.630	-1.391253	-1.537563	-1.387427	-1.494365	-1.385674	-1.478058	-2.182235
1.635	-1.378027	-1.526072	-1.374327	-1.482658	-1.372607	-1.466425	-2.176384
1.640	-1.364831	-1.514616	-1.361268	-1.470897	-1.359500	-1.454781	-2.170419
1.645	-1.351459	-1.502962	-1.348019	-1.459055	-1.346395	-1.443137	-2.164400
1.650	-1.338216	-1.491455	-1.334922	-1.447161	-1.333144	-1.431394	-2.158239
1.655	-1.324875	-1.479824	-1.321712	-1.435210	-1.319932	-1.419691	-2.152013
1.660	-1.311506	-1.468156	-1.308474	-1.423203	-1.306690	-1.407976	-2.145700
1.665	-1.298115	-1.456455	-1.295213	-1.411145	-1.293426	-1.396256	-2.139302
1.670	-1.284705	-1.444725	-1.281932	-1.399037	-1.280142	-1.384532	-2.132819
1.675	-1.271277	-1.432966	-1.268633	-1.386883	-1.266839	-1.372807	-2.126253
1.680	-1.257758	-1.421085	-1.255226	-1.374683	-1.253588	-1.361132	-2.119635
1.685	-1.244363	-1.409354	-1.241974	-1.362438	-1.240179	-1.349349	-2.112884
1.690	-1.230911	-1.397530	-1.228646	-1.350162	-1.226848	-1.337643	-2.106081
1.695	-1.217362	-1.385582	-1.215206	-1.337844	-1.213567	-1.325987	-2.099233
1.700	-1.203930	-1.373757	-1.201901	-1.325503	-1.200216	-1.314301	-2.092270
1.705	-1.190408	-1.361822	-1.188498	-1.313112	-1.186859	-1.302610	-2.085259
1.710	-1.176932	-1.349921	-1.175143	-1.300699	-1.173503	-1.290942	-2.078167
1.715	-1.163444	-1.338032	-1.161806	-1.288240	-1.160038	-1.279206	-2.070977
1.720	-1.150049	-1.326169	-1.148521	-1.275792	-1.146702	-1.267585	-2.063745

Continued on next page

Table S3 – Continued from previous page

r(Å)	pCCD(OO)	CCD(OO)	CCSD(OO)	fpCCD(OO)	fpCCSD(OO)	fpLCCD(OO)	fpLCCSD(OO)
1.725	-1.136534	-1.314164	-1.135105	-1.263304	-1.133430	-1.256024	-2.056482
1.730	-1.123253	-1.302431	-1.121975	-1.250827	-1.120016	-1.244375	-2.049097
1.735	-1.109812	-1.290484	-1.108651	-1.238314	-1.106654	-1.232770	-2.041698
1.740	-1.096399	-1.278555	-1.095359	-1.225783	-1.093378	-1.221287	-2.034214
1.745	-1.082931	-1.266495	-1.081960	-1.213249	-1.080280	-1.209945	-2.026657
1.750	-1.069731	-1.254770	-1.068900	-1.200722	-1.066992	-1.198499	-2.019019
1.755	-1.056195	-1.242613	-1.055440	-1.188151	-1.053824	-1.187093	-2.011401
1.760	-1.042908	-1.230800	-1.042324	-1.175571	-1.040412	-1.175551	-2.003690
1.765	-1.029751	-1.219011	-1.029259	-1.163044	-1.027394	-1.164328	-1.995890
1.770	-1.016348	-1.206936	-1.015936	-1.150479	-1.014255	-1.153051	-1.988093
1.775	-1.003281	-1.195214	-1.002974	-1.137958	-1.001222	-1.141900	-1.980209
1.780	-0.990011	-1.183325	-0.989863	-1.125377	-0.988001	-1.130534	-1.972299
1.785	-0.976710	-1.171293	-0.976638	-1.112828	-0.974952	-1.119371	-1.964322
1.790	-0.963665	-1.159531	-0.963695	-1.100314	-0.961976	-1.108299	-1.956305
1.795	-0.950592	-1.147726	-0.950730	-1.087785	-0.948949	-1.097175	-1.948247
1.800	-0.937596	-1.135964	-0.937826	-1.075286	-0.936041	-1.086181	-1.940160
1.805	-0.924593	-1.124187	-0.924926	-1.062799	-0.923179	-1.075247	-1.932036
1.810	-0.911669	-1.112475	-0.912106	-1.050313	-0.910301	-1.064304	-1.923868
1.815	-0.898845	-1.100842	-0.899384	-1.037859	-0.897494	-1.053458	-1.915658
1.820	-0.885754	-1.088892	-0.886379	-1.025386	-0.884688	-1.042558	-1.907464
1.825	-0.873016	-1.077335	-0.873767	-1.012958	-0.871889	-1.031750	-1.899183
1.830	-0.860035	-1.065479	-0.860894	-1.000525	-0.859122	-1.020935	-1.890899
1.835	-0.847207	-1.053730	-0.848146	-0.988135	-0.846455	-1.010236	-1.882599
1.840	-0.834503	-1.042112	-0.835544	-0.975762	-0.833793	-0.999531	-1.874254
1.845	-0.821741	-1.030424	-0.822893	-0.963418	-0.821199	-0.988918	-1.865908
1.850	-0.809076	-1.018810	-0.810337	-0.951101	-0.808624	-0.978334	-1.857525
1.855	-0.796516	-1.007273	-0.797877	-0.938818	-0.796100	-0.967807	-1.849116
1.860	-0.783963	-0.995722	-0.785426	-0.926566	-0.783639	-0.957343	-1.840693
1.865	-0.771460	-0.984197	-0.773023	-0.914349	-0.771233	-0.946935	-1.832264
1.870	-0.759011	-0.972703	-0.760673	-0.902168	-0.758882	-0.936585	-1.823805
1.875	-0.746553	-0.961167	-0.748307	-0.890024	-0.746603	-0.926302	-1.815341
1.880	-0.734296	-0.949823	-0.736143	-0.877931	-0.734411	-0.916121	-1.806859
1.885	-0.722018	-0.938438	-0.723966	-0.865868	-0.722225	-0.905945	-1.798361
1.890	-0.709801	-0.927092	-0.711851	-0.853848	-0.710097	-0.895829	-1.789851
1.895	-0.697646	-0.915786	-0.699797	-0.841873	-0.698030	-0.885776	-1.781332
1.900	-0.685523	-0.904497	-0.687786	-0.829936	-0.686000	-0.875743	-1.772825
1.905	-0.673485	-0.893258	-0.675845	-0.818055	-0.674062	-0.865814	-1.764296
1.910	-0.661467	-0.882017	-0.663928	-0.806225	-0.662190	-0.855960	-1.755762
1.915	-0.649742	-0.871033	-0.652277	-0.794456	-0.650419	-0.846210	-1.747226
1.920	-0.637767	-0.859760	-0.640383	-0.782729	-0.638692	-0.836472	-1.738682
1.925	-0.626174	-0.848924	-0.628918	-0.771060	-0.627083	-0.826891	-1.730144
1.930	-0.614358	-0.837732	-0.617151	-0.759445	-0.615495	-0.817257	-1.721592
1.935	-0.602814	-0.826904	-0.605746	-0.747881	-0.603920	-0.807716	-1.713040
1.940	-0.591097	-0.815820	-0.594133	-0.736372	-0.592429	-0.798189	-1.704524
1.945	-0.579622	-0.804932	-0.582728	-0.724935	-0.581090	-0.788825	-1.695991
1.950	-0.568373	-0.794327	-0.571607	-0.713553	-0.569809	-0.779558	-1.687457
1.955	-0.556985	-0.783462	-0.560282	-0.702234	-0.558555	-0.770246	-1.678934
1.960	-0.545732	-0.772784	-0.549145	-0.690981	-0.547396	-0.761070	-1.670412
1.965	-0.534519	-0.762078	-0.538017	-0.679797	-0.536348	-0.751967	-1.661909
1.970	-0.523502	-0.751538	-0.527064	-0.668688	-0.525414	-0.743018	-1.653392

Continued on next page

Table S3 – Continued from previous page

r(Å)	pCCD(OO)	CCD(OO)	CCSD(OO)	fpCCD(OO)	fpCCSD(OO)	fpLCCD(OO)	fpLCCSD(OO)
1.975	-0.512431	-0.740957	-0.516104	-0.657628	-0.514475	-0.734010	-1.644907
1.980	-0.501440	-0.730476	-0.505266	-0.646624	-0.503577	-0.725019	-1.636450
1.985	-0.490764	-0.720240	-0.494669	-0.635717	-0.492922	-0.716338	-1.627958
1.990	-0.479993	-0.709882	-0.483995	-0.624870	-0.482257	-0.707591	-1.619501
1.995	-0.469192	-0.699454	-0.473291	-0.614092	-0.471583	-0.698788	-1.611068
2.000	-0.458647	-0.689253	-0.462813	-0.603406	-0.461185	-0.690294	-1.602649
2.005	-0.448068	-0.679043	-0.452361	-0.592763	-0.450675	-0.681658	-1.594224
2.010	-0.437634	-0.668938	-0.442030	-0.582208	-0.440353	-0.673208	-1.585834
2.015	-0.427287	-0.658888	-0.431777	-0.571733	-0.430106	-0.664823	-1.577455
2.020	-0.417026	-0.648894	-0.421603	-0.561336	-0.419958	-0.656533	-1.569096
2.025	-0.406850	-0.638954	-0.411508	-0.551019	-0.409925	-0.648322	-1.560728
2.030	-0.396792	-0.629123	-0.401541	-0.540774	-0.399937	-0.640153	-1.552419
2.035	-0.386802	-0.619348	-0.391649	-0.530608	-0.390042	-0.632067	-1.544109
2.040	-0.376928	-0.609633	-0.381835	-0.520538	-0.380262	-0.624087	-1.535858
2.045	-0.367010	-0.599928	-0.372071	-0.510516	-0.370500	-0.616096	-1.527595
2.050	-0.357308	-0.590418	-0.362501	-0.500582	-0.360892	-0.608245	-1.519361
2.055	-0.347718	-0.580936	-0.352984	-0.490749	-0.351375	-0.600510	-1.511160
2.060	-0.338163	-0.571481	-0.343523	-0.480993	-0.341934	-0.592793	-1.502972
2.065	-0.328724	-0.562070	-0.334130	-0.471329	-0.332651	-0.585193	-1.494808
2.070	-0.319275	-0.552771	-0.324867	-0.461721	-0.323284	-0.577542	-1.486699
2.075	-0.310057	-0.543530	-0.315682	-0.452227	-0.314175	-0.570109	-1.478591
2.080	-0.300858	-0.534349	-0.306578	-0.442806	-0.305090	-0.562684	-1.470505
2.085	-0.291766	-0.525302	-0.297626	-0.433459	-0.296084	-0.555308	-1.462469
2.090	-0.282814	-0.516358	-0.288788	-0.424198	-0.287201	-0.548068	-1.454459
2.095	-0.273915	-0.507424	-0.279989	-0.415032	-0.278410	-0.540879	-1.446472
2.100	-0.265111	-0.498569	-0.271288	-0.405952	-0.269716	-0.533768	-1.438512
2.105	-0.256402	-0.489790	-0.262682	-0.396958	-0.261114	-0.526729	-1.430584
2.110	-0.247757	-0.480982	-0.254079	-0.388078	-0.252632	-0.519778	-1.422685
2.115	-0.239297	-0.472468	-0.245758	-0.379238	-0.244233	-0.512901	-1.414831
2.120	-0.230879	-0.463918	-0.237438	-0.370507	-0.235913	-0.506083	-1.406998
2.125	-0.222527	-0.455418	-0.229190	-0.361868	-0.227683	-0.499330	-1.399200
2.130	-0.214247	-0.446912	-0.220970	-0.353336	-0.219529	-0.492631	-1.391423
2.135	-0.206117	-0.438609	-0.212950	-0.344862	-0.211500	-0.486018	-1.383696
2.140	-0.198048	-0.430311	-0.204961	-0.336503	-0.203556	-0.479517	-1.376003
2.145	-0.189994	-0.422097	-0.197076	-0.328210	-0.195682	-0.473013	-1.368332
2.150	-0.182192	-0.414045	-0.189355	-0.320007	-0.187952	-0.466652	-1.360707
2.155	-0.174435	-0.406025	-0.181693	-0.311899	-0.180295	-0.460335	-1.353118
2.160	-0.166764	-0.398074	-0.174117	-0.303882	-0.172737	-0.454093	-1.345549
2.165	-0.159185	-0.390186	-0.166624	-0.295959	-0.165250	-0.447918	-1.338027
2.170	-0.151698	-0.382385	-0.159232	-0.288122	-0.157871	-0.441817	-1.330537
2.175	-0.144274	-0.374717	-0.151984	-0.280351	-0.150576	-0.435747	-1.323116
2.180	-0.136995	-0.367025	-0.144738	-0.272711	-0.143395	-0.429812	-1.315680
2.185	-0.129758	-0.359514	-0.137680	-0.265117	-0.136292	-0.423884	-1.308319
2.190	-0.122663	-0.352007	-0.130645	-0.257642	-0.129285	-0.418063	-1.300978
2.195	-0.115638	-0.344609	-0.123733	-0.250245	-0.122358	-0.412291	-1.293688
2.200	-0.108705	-0.337275	-0.116903	-0.242942	-0.115544	-0.406599	-1.286422
2.205	-0.101867	-0.330016	-0.110164	-0.235730	-0.108829	-0.400979	-1.279202
2.210	-0.095121	-0.322836	-0.103518	-0.228605	-0.102204	-0.395424	-1.272028
2.215	-0.088470	-0.315710	-0.096944	-0.221581	-0.095657	-0.389941	-1.264868
2.220	-0.081839	-0.308681	-0.090483	-0.214627	-0.089183	-0.384482	-1.257783

Continued on next page

Table S3 – Continued from previous page

r(Å)	pCCD(OO)	CCD(OO)	CCSD(OO)	fpCCD(OO)	fpCCSD(OO)	fpLCCD(OO)	fpLCCSD(OO)
2.225	-0.075441	-0.301779	-0.084146	-0.207757	-0.082852	-0.379131	-1.250712
2.230	-0.069060	-0.294917	-0.077873	-0.200982	-0.076580	-0.373830	-1.243689
2.235	-0.062767	-0.288127	-0.071685	-0.194297	-0.070404	-0.368598	-1.236704
2.240	-0.056563	-0.281386	-0.065561	-0.187716	-0.064323	-0.363468	-1.229740
2.245	-0.050424	-0.274742	-0.059550	-0.181205	-0.058332	-0.358350	-1.222855
2.250	-0.044402	-0.268225	-0.053674	-0.174765	-0.052406	-0.353283	-1.215990
2.255	-0.038473	-0.261727	-0.047825	-0.168431	-0.046605	-0.348313	-1.209168
2.260	-0.032628	-0.255317	-0.042080	-0.162188	-0.040899	-0.343425	-1.202390
2.265	-0.026892	-0.249044	-0.036474	-0.156000	-0.035207	-0.338536	-1.195622
2.270	-0.021207	-0.242727	-0.030857	-0.149951	-0.029681	-0.333774	-1.188927
2.275	-0.015612	-0.236581	-0.025415	-0.144036	-0.024179	-0.328978	-1.182270
2.280	-0.010122	-0.230445	-0.019988	-0.138145	-0.018834	-0.324363	-1.175646
2.285	-0.004691	-0.224447	-0.014717	-0.132309	-0.013530	-0.319716	-1.169073
2.290	0.000620	-0.218464	-0.009465	-0.126587	-0.008332	-0.315190	-1.162530
2.295	0.005887	-0.212579	-0.004333	-0.120939	-0.003204	-0.310661	-1.156047
2.300	0.011005	-0.206879	0.000640	-0.115319	0.001831	-0.306239	-1.149591
2.305	0.016092	-0.201058	0.005679	-0.109876	0.006782	-0.301870	-1.143163
2.310	0.021082	-0.195503	0.010480	-0.104421	0.011692	-0.297521	-1.136756
2.315	0.025990	-0.189823	0.015357	-0.099148	0.016439	-0.293276	-1.130459
2.320	0.030797	-0.184354	0.020050	-0.093879	0.021114	-0.289078	-1.124169
2.325	0.035567	-0.178964	0.024641	-0.088702	0.025761	-0.284898	-1.117939
2.330	0.040229	-0.173576	0.029205	-0.083638	0.030301	-0.280784	-1.111731
2.335	0.044781	-0.168295	0.033687	-0.078629	0.034753	-0.276773	-1.105553
2.340	0.049316	-0.163078	0.038070	-0.073706	0.039164	-0.272748	-1.099429
2.345	0.053670	-0.157931	0.042397	-0.068867	0.043446	-0.268860	-1.093314
2.350	0.058073	-0.152816	0.046650	-0.064125	0.047689	-0.264920	-1.087289
2.355	0.062324	-0.147868	0.050763	-0.059408	0.051836	-0.261081	-1.081288
2.360	0.066531	-0.142884	0.054876	-0.054827	0.055867	-0.257310	-1.075341
2.365	0.070619	-0.138090	0.058823	-0.050258	0.059892	-0.253547	-1.069423
2.370	0.074679	-0.133271	0.062766	-0.045812	0.063804	-0.249880	-1.063526
2.375	0.078639	-0.128535	0.066620	-0.041441	0.067633	-0.246245	-1.057702
2.380	0.082502	-0.123868	0.070405	-0.037137	0.071382	-0.242667	-1.051914
2.385	0.086321	-0.119260	0.074116	-0.032924	0.075093	-0.239121	-1.046149
2.390	0.090067	-0.114785	0.077704	-0.028742	0.078699	-0.235637	-1.040444
2.395	0.093661	-0.110375	0.081255	-0.024612	0.082206	-0.232273	-1.034744
2.400	0.097331	-0.105927	0.084756	-0.020652	0.085712	-0.228821	-1.029133
2.405	0.100824	-0.101625	0.088167	-0.016691	0.089079	-0.225520	-1.023547
2.410	0.104250	-0.097395	0.091468	-0.012821	0.092454	-0.222158	-1.017986
2.415	0.107657	-0.093248	0.094718	-0.008983	0.095675	-0.218959	-1.012474
2.420	0.110915	-0.089112	0.097944	-0.005252	0.098852	-0.215787	-1.006990
2.425	0.114191	-0.084963	0.101111	-0.001679	0.102000	-0.212565	-1.001575
2.430	0.117364	-0.081003	0.104142	0.001936	0.105038	-0.209438	-0.996184
2.435	0.120489	-0.077090	0.107132	0.005479	0.108038	-0.206377	-0.990824
2.440	0.123494	-0.073278	0.110015	0.008971	0.110948	-0.203355	-0.985525
2.445	0.126489	-0.069439	0.112901	0.012335	0.113813	-0.200368	-0.980249
2.450	0.129391	-0.065669	0.115714	0.015638	0.116563	-0.197441	-0.975025
2.455	0.132234	-0.062004	0.118430	0.018905	0.119304	-0.194523	-0.969821
2.460	0.135018	-0.058401	0.121082	0.022111	0.121963	-0.191650	-0.964671
2.465	0.137724	-0.054825	0.123698	0.025229	0.124546	-0.188857	-0.959558
2.470	0.140376	-0.051342	0.126224	0.028304	0.127090	-0.186066	-0.954480

Continued on next page

Table S3 – Continued from previous page

r(Å)	pCCD(OO)	CCD(OO)	CCSD(OO)	fpCCD(OO)	fpCCSD(OO)	fpLCCD(OO)	fpLCCSD(OO)
2.475	0.142971	-0.047912	0.128694	0.031314	0.129553	-0.183322	-0.949444
2.480	0.145497	-0.044531	0.131111	0.034255	0.131965	-0.180628	-0.944438
2.485	0.147961	-0.041207	0.133468	0.037133	0.134310	-0.177977	-0.939486
2.490	0.150359	-0.037934	0.135772	0.039946	0.136586	-0.175367	-0.934565
2.495	0.152721	-0.034748	0.137995	0.042723	0.138815	-0.172788	-0.929687
2.500	0.155008	-0.031586	0.140187	0.045419	0.140987	-0.170256	-0.924833
2.505	0.157255	-0.028463	0.142331	0.048048	0.143104	-0.167726	-0.920025
2.510	0.159394	-0.025428	0.144404	0.050631	0.145152	-0.165312	-0.915236
2.515	0.161512	-0.022492	0.146376	0.053195	0.147147	-0.162866	-0.910518
2.520	0.163565	-0.019537	0.148346	0.055639	0.149101	-0.160469	-0.905807
2.525	0.165588	-0.016697	0.150221	0.058084	0.150985	-0.158109	-0.901155
2.530	0.167543	-0.013871	0.152066	0.060435	0.152814	-0.155783	-0.896543
2.535	0.169446	-0.011109	0.153853	0.062744	0.154600	-0.153492	-0.891951
2.540	0.171296	-0.008395	0.155590	0.064995	0.156329	-0.151238	-0.887402
2.545	0.173096	-0.005733	0.157278	0.067197	0.158004	-0.149021	-0.882889
2.550	0.174843	-0.003122	0.158914	0.069345	0.159629	-0.146836	-0.878413
2.555	0.176538	-0.000562	0.160495	0.071436	0.161202	-0.144680	-0.873975
2.560	0.178177	0.001952	0.162022	0.073461	0.162722	-0.142548	-0.869580
2.565	0.179793	0.004430	0.163507	0.075420	0.164189	-0.140454	-0.865216
2.570	0.181338	0.006856	0.164953	0.077345	0.165613	-0.138394	-0.860881
2.575	0.182835	0.009220	0.166339	0.079234	0.166992	-0.136365	-0.856584
2.580	0.184286	0.011529	0.167674	0.081078	0.168319	-0.134371	-0.852334
2.585	0.185690	0.013790	0.168961	0.082874	0.169599	-0.132408	-0.848105
2.590	0.187048	0.016008	0.170205	0.084619	0.170840	-0.130470	-0.843914
2.595	0.188358	0.018177	0.171402	0.086319	0.172035	-0.128560	-0.839757
2.600	0.189624	0.020304	0.172556	0.087969	0.173183	-0.126680	-0.835635
2.605	0.190854	0.022386	0.173670	0.089576	0.174290	-0.124831	-0.831541
2.610	0.192033	0.024423	0.174736	0.091134	0.175351	-0.123009	-0.827487
2.615	0.193164	0.026412	0.175755	0.092648	0.176370	-0.121213	-0.823467
2.620	0.194256	0.028353	0.176735	0.094133	0.177353	-0.119448	-0.819474
2.625	0.195299	0.030236	0.177668	0.095597	0.178289	-0.117728	-0.815521
2.630	0.196351	0.032112	0.178592	0.096995	0.179190	-0.116025	-0.811578
2.635	0.197332	0.033914	0.179439	0.098360	0.180070	-0.114329	-0.807695
2.640	0.198219	0.035721	0.180286	0.099641	0.180850	-0.112686	-0.803849
2.645	0.199086	0.037423	0.181023	0.100896	0.181618	-0.111058	-0.800032
2.650	0.200007	0.039132	0.181794	0.102154	0.182363	-0.109460	-0.796238
2.655	0.200786	0.040818	0.182515	0.103296	0.183080	-0.107871	-0.792463
2.660	0.201598	0.042419	0.183181	0.104478	0.183747	-0.106319	-0.788739
2.665	0.202348	0.044014	0.183838	0.105593	0.184361	-0.104812	-0.785044
2.670	0.203033	0.045545	0.184420	0.106654	0.184956	-0.103288	-0.781391
2.675	0.203695	0.047049	0.184971	0.107661	0.185501	-0.101806	-0.777759
2.680	0.204361	0.048495	0.185491	0.108694	0.186039	-0.100349	-0.774153
2.685	0.204949	0.049920	0.186003	0.109614	0.186504	-0.098926	-0.770586
2.690	0.205533	0.051274	0.186457	0.110614	0.186995	-0.097506	-0.767027
2.695	0.206041	0.052662	0.186921	0.111493	0.187403	-0.096147	-0.763524
2.700	0.206552	0.053975	0.187296	0.112327	0.187794	-0.094770	-0.760055
2.705	0.207012	0.055269	0.187664	0.113133	0.188131	-0.093428	-0.756605
2.710	0.207477	0.056484	0.187967	0.113953	0.188492	-0.092072	-0.753172
2.715	0.207915	0.057733	0.188297	0.114683	0.188756	-0.090814	-0.749804
2.720	0.208241	0.058911	0.188567	0.115413	0.189020	-0.089528	-0.746440

Continued on next page

Table S3 – Continued from previous page

r(Å)	pCCD(OO)	CCD(OO)	CCSD(OO)	fpCCD(OO)	fpCCSD(OO)	fpLCCD(OO)	fpLCCSD(OO)
2.725	0.208579	0.060054	0.188796	0.116097	0.189258	-0.088262	-0.743128
2.730	0.208938	0.061164	0.189000	0.116762	0.189468	-0.087017	-0.739822
2.735	0.209200	0.062253	0.189197	0.117406	0.189645	-0.085811	-0.736549
2.740	0.209489	0.063295	0.189341	0.118012	0.189800	-0.084607	-0.733308
2.745	0.209735	0.064321	0.189474	0.118581	0.189923	-0.083430	-0.730094
2.750	0.209945	0.065311	0.189575	0.119127	0.190020	-0.082273	-0.726909
2.755	0.210128	0.066271	0.189651	0.119644	0.190090	-0.081135	-0.723752
2.760	0.210285	0.067202	0.189700	0.120133	0.190135	-0.080016	-0.720624
2.765	0.210416	0.068103	0.189724	0.120595	0.190154	-0.078915	-0.717523
2.770	0.210508	0.068961	0.189707	0.121028	0.190155	-0.077819	-0.714448
2.775	0.210619	0.069826	0.189707	0.121445	0.190119	-0.076779	-0.711393
2.780	0.210686	0.070630	0.189650	0.121843	0.190081	-0.075724	-0.708381
2.785	0.210700	0.071425	0.189581	0.122187	0.189991	-0.074697	-0.705392
2.790	0.210723	0.072186	0.189501	0.122553	0.189905	-0.073690	-0.702414
2.795	0.210662	0.072909	0.189354	0.122818	0.189766	-0.072680	-0.699473
2.800	0.210670	0.073621	0.189253	0.123187	0.189665	-0.071716	-0.696564
2.805	0.210614	0.074304	0.189091	0.123447	0.189500	-0.070755	-0.693679
2.810	0.210540	0.074962	0.188909	0.123687	0.189315	-0.069811	-0.690817
2.815	0.210445	0.075595	0.188707	0.123904	0.189109	-0.068883	-0.687982
2.820	0.210329	0.076204	0.188485	0.124099	0.188883	-0.067972	-0.685173
2.825	0.210193	0.076788	0.188242	0.124271	0.188638	-0.067075	-0.682388
2.830	0.210038	0.077348	0.187980	0.124423	0.188373	-0.066194	-0.679629
2.835	0.209856	0.077913	0.187701	0.124482	0.188067	-0.065332	-0.676920
2.840	0.209643	0.078411	0.187392	0.124613	0.187766	-0.064488	-0.674206
2.845	0.209467	0.078887	0.187078	0.124758	0.187460	-0.063666	-0.671520
2.850	0.209216	0.079379	0.186744	0.124772	0.187093	-0.062833	-0.668863
2.855	0.208970	0.079835	0.186401	0.124817	0.186744	-0.062029	-0.666234
2.860	0.208704	0.080265	0.186039	0.124856	0.186377	-0.061238	-0.663608
2.865	0.208410	0.080681	0.185661	0.124854	0.185981	-0.060464	-0.661042
2.870	0.208130	0.081057	0.185255	0.124867	0.185578	-0.059700	-0.658470
2.875	0.207813	0.081383	0.184815	0.124906	0.185192	-0.058916	-0.655932
2.880	0.207460	0.081755	0.184381	0.124777	0.184713	-0.058197	-0.653451
2.885	0.207182	0.082058	0.183933	0.124794	0.184275	-0.057481	-0.650950
2.890	0.206767	0.082377	0.183468	0.124673	0.183812	-0.056758	-0.648479
2.895	0.206392	0.082682	0.183009	0.124574	0.183330	-0.056064	-0.646048
2.900	0.206024	0.082966	0.182549	0.124502	0.182839	-0.055403	-0.643604
2.905	0.205614	0.083199	0.182033	0.124395	0.182347	-0.054707	-0.641211
2.910	0.205194	0.083428	0.181510	0.124257	0.181831	-0.054041	-0.638849
2.915	0.204831	0.083645	0.180978	0.124113	0.181298	-0.053405	-0.636499
2.920	0.204344	0.083849	0.180430	0.123911	0.180739	-0.052764	-0.634158
2.925	0.203871	0.084014	0.179862	0.123750	0.180195	-0.052125	-0.631844
2.930	0.203426	0.084201	0.179317	0.123546	0.179615	-0.051529	-0.629565
2.935	0.202957	0.084353	0.178747	0.123352	0.179041	-0.050926	-0.627293
2.940	0.202451	0.084495	0.178162	0.123109	0.178446	-0.050342	-0.625062
2.945	0.201998	0.084661	0.177645	0.122942	0.177910	-0.049759	-0.622789
2.950	0.201492	0.084732	0.177001	0.122714	0.177265	-0.049193	-0.620626
2.955	0.200969	0.084856	0.176417	0.122435	0.176641	-0.048651	-0.618451
2.960	0.200500	0.084888	0.175761	0.122243	0.176036	-0.048093	-0.616256
2.965	0.199881	0.084948	0.175083	0.121852	0.175360	-0.047535	-0.614147
2.970	0.199342	0.084995	0.174444	0.121583	0.174706	-0.047019	-0.612033

Continued on next page

Table S3 – Continued from previous page

r(Å)	pCCD(OO)	CCD(OO)	CCSD(OO)	fpCCD(OO)	fpCCSD(OO)	fpLCCD(OO)	fpLCCSD(OO)
2.975	0.198763	0.085008	0.173765	0.121282	0.174056	-0.046484	-0.609927
2.980	0.198207	0.085009	0.173084	0.120985	0.173379	-0.045989	-0.607853
2.985	0.197615	0.085058	0.172454	0.120642	0.172723	-0.045480	-0.605801
2.990	0.197092	0.085023	0.171763	0.120384	0.172056	-0.045002	-0.603736
2.995	0.196455	0.085037	0.171092	0.119979	0.171348	-0.044518	-0.601734
3.000	0.195878	0.085009	0.170396	0.119623	0.170633	-0.044065	-0.599746
