# Supplementary material for: Expectation Value-pCCD-Based Methods for Single-Electron Properties
Source: J Phys Chem A. 2025 Jul 16;129(29):6713–32. doi: 10.1021/acs.jpca.5c03859 (PMC12302219; doi:10.1021/acs.jpca.5c03859)
Supplement: Supplementary file 3 [file jp5c03859_si_003.pdf]

Expectation Value-pCCD-based Methods for Single-electron Properties

Rahul Chakraborty, Somayeh Ahmadkhani*, Julian Świerczyński,
Katharina Boguslawski, and Paweł Tecmer*

Institute of Physics, Faculty of Physics, Astronomy, and Informatics
Nicolaus Copernicus University in Toruń,
Grudziadzka 5, 87-100 Toruń, Poland

Corresponding authors: so.ahmadkhani@gmail.com, ptecmer@fizyka.umk.pl

Section I. Bond lengths of diatomic molecules and ions

Molecule/Ion	Bond length (Å)
H_2	0.741
HF	0.917
HCl	1.275
LiH	1.596
NaH	1.889
LiF	1.564
NaF	1.926
NaCl	2.361
ClF	1.628
LiNa	2.810
N_2	1.098
CN^-	1.172
NO^+	1.060
PN	1.491
CO	1.128
CS	1.535
CSe	1.676
SiO	1.510
SiS	1.730
SiSe	2.058
GeO	1.625
GeS	2.012
GeSe	2.135

Section II. Coordinates of small polyatomic molecules

All coordinates are given in Å.

Molecule: CO_2

Atom	X	Y	Z
C	0.0000	0.0000	0.0000
O	0.0000	0.0000	1.1621
O	0.0000	0.0000	-1.1621

Molecule: H_2O

Atom	X	Y	Z
O	0.0000	0.0000	0.1173
H	0.0000	0.7572	-0.4692
H	0.0000	-0.7572	-0.4692

Molecule: NH_3

Atom	X	Y	Z
N	0.0000	0.0000	0.0000
H	0.0000	-0.9377	-0.3816
H	0.8121	0.4689	-0.3816
H	-0.8121	0.4689	-0.3816

Molecule: PH_3

Atom	X	Y	Z
P	0.0000	0.0000	0.0000
H	0.0000	-1.1932	-0.7717
H	1.033	0.5966	-0.7717
H	-1.033	0.5966	-0.7717

Note: Molecules/ions in Section I and Section II constitute the 'small' set investigated in this work.

Section III. Coordinates of organic molecules

All coordinates are given in Å.

Molecule: HCCF

Atom	X	Y	Z
C	0.0000	0.0000	-0.0942
C	0.0000	0.0000	-1.2922
F	0.0000	0.0000	1.18480
H	0.0000	0.0000	-2.3452

Molecule: 1-Propanol

Atom	X	Y	Z
C	-0.558259	0.106999	-0.098372
C	0.039544	0.106999	-1.494530
C	0.555103	0.000000	0.929051
H	-0.748484	0.182733	-2.221730
H	0.584826	-0.806118	-1.650840
H	0.705411	0.944383	-1.599210
H	-1.224130	-0.730385	0.006305
H	-1.106540	1.025140	0.058797
H	1.100390	-0.913117	0.772742
H	1.220970	0.837384	0.824374
H	-0.568829	-0.754021	2.247920
O	0.000000	0.000000	2.225480

Molecule: 1-Methylpropane

Atom	X	Y	Z
C	0.000000	-1.431900	0.072322
C	0.000000	0.000000	-0.433930
C	1.240060	0.715949	0.072322
C	-1.240060	0.715949	0.072322
H	0.000000	0.000000	-1.508890
H	0.000000	-1.431900	1.147290
H	0.877704	-1.938640	-0.286000
H	-0.877704	-1.938640	-0.286000
H	1.240060	0.715949	1.147290
H	-1.240060	0.715949	1.147290
H	1.240060	1.729430	-0.286000
H	-1.240060	1.729430	-0.286000
H	2.117760	0.209206	-0.286000
H	-2.117760	0.209206	-0.286000

Molecule: 2-Nitropropane

Atom	X	Y	Z
C	-0.584385	-0.098193	1.240060
C	-0.584385	-0.098193	-1.240060
C	0.251851	-0.361974	0.000000
H	-1.440300	-0.748539	1.240060
H	-1.440300	-0.748539	-1.240060
H	-0.912231	0.925557	1.240060
H	-0.912231	0.925557	-1.240060
H	0.007497	-0.284897	2.117760
H	0.007497	-0.284897	-2.117760
H	0.579697	-1.385720	0.000000
O	1.197330	1.686510	0.000000
O	2.478770	0.000000	0.000000
N	1.351200	0.473333	0.000000

Molecule: 2-propanol

Atom	X	Y	Z
C	0.003953	-0.381184	0.285277
C	-1.123380	0.329382	-0.443316
C	1.332750	0.000000	-0.343695
H	-2.063890	0.059583	0.001865
H	-1.120360	0.038829	-1.478260
H	-0.983786	1.392670	-0.369242
H	-0.135636	-1.444470	0.211204
H	1.335770	-0.290553	-1.378640
H	2.130670	-0.502933	0.171998
H	1.472340	1.063290	-0.269621
H	-0.848435	-0.264607	1.969380
O	0.000000	0.000000	1.643050

Molecule: 3-Pentanone

Atom	X	Y	Z
C	1.298200	0.000000	-0.595169
C	-1.298200	0.000000	-0.595169
C	0.000000	0.000000	0.154347
C	2.452580	0.000000	0.391764
C	-2.452580	0.000000	0.391764
H	1.355140	0.877704	-1.213180
H	-1.355140	0.877704	-1.213180
H	1.355140	-0.877704	-1.213180
H	-1.355140	-0.877704	-1.213180
H	2.395630	0.877704	1.009780
H	-2.395630	0.877704	1.009780
H	2.395630	-0.877704	1.009780
H	-2.395630	-0.877704	1.009780
H	3.383520	0.000000	-0.145718
H	-3.383520	0.000000	-0.145718
O	0.000000	0.000000	1.357520

Molecule: C_2H_2

Atom	X	Y	Z
C	0.0000	0.0000	0.6013
C	0.0000	0.0000	-0.6013
H	0.0000	0.0000	1.6644
H	0.0000	0.0000	-1.6644

Molecule: CH_3CCH

Atom	X	Y	Z
C	0.0000	0.0000	-1.2455
C	0.0000	0.0000	0.2135
C	0.0000	0.0000	1.4195
H	0.0000	0.0000	2.4755
H	0.0000	1.0465	-1.6003
H	0.9063	-0.5232	-1.6003
H	-0.9063	-0.5232	-1.6003

Molecule: CH_3F

Atom	X	Y	Z
C	0.0000	0.0000	-0.6244
F	0.0000	0.0000	0.7402
H	0.0000	1.0245	-0.9718
H	0.8873	-0.5123	-0.9718
H	-0.8873	-0.5123	-0.9718

Molecule: Acetaldehyde

Atom	X	Y	Z
C	0.000000	0.503552	0.682688
C	0.000000	-0.361795	-0.541352
H	0.000000	-1.396210	-0.248885
H	0.877704	-0.154860	-1.126470
H	-0.877704	-0.154860	-1.126470

H	0.000000	1.564170	0.585068
O	0.000000	0.000000	1.775420

Molecule: Acetone

Atom	X	Y	Z
C	0.000000	1.298200	0.162723
C	0.000000	-1.298200	0.162723
C	0.000000	0.000000	-0.586792
H	0.000000	2.115260	-0.535819
H	0.000000	-2.115260	-0.535819
H	0.877704	1.355140	0.780737
H	0.877704	-1.355140	0.780737
H	-0.877704	1.355140	0.780737
H	-0.877704	-1.355140	0.780737
O	0.000000	0.000000	-1.789960

Molecule: Aminopropane

Atom	X	Y	Z
C	0.113302	0.426103	0.000000
C	-0.581020	-0.109424	1.240060
C	-0.581020	-0.109424	-1.240060
H	0.068692	1.500140	0.000000
H	-0.536410	-1.183460	1.240060
H	-0.536410	-1.183460	-1.240060
H	-1.608500	0.206530	1.240060
H	-1.608500	0.206530	-1.240060
H	-0.089584	0.269619	2.117760
H	-0.089584	0.269619	-2.117760
H	1.721480	-0.970900	0.000000
H	2.228570	0.678136	0.000000
N	1.498990	0.000000	0.000000

Molecule: Benzene

Atom	X	Y	Z
C	1.380710	0.000000	0.000000
C	0.690369	-1.195730	0.000000
C	0.690369	1.195730	0.000000
C	-0.690369	-1.195730	0.000000
C	-0.690369	1.195730	0.000000
C	-1.380710	0.000000	0.000000
H	2.449790	0.000000	0.000000
H	1.224900	-2.121580	0.000000
H	1.224900	2.121580	0.000000
H	-1.224900	-2.121580	0.000000
H	-1.224900	2.121580	0.000000
H	-2.449790	0.000000	0.000000

Molecule: Chlorobenzene

Atom	X	Y	Z
C	0.000000	0.000000	1.380750
C	-1.195760	0.000000	0.690374
C	1.195760	0.000000	0.690374
C	-1.195760	0.000000	-0.690374
C	1.195760	0.000000	-0.690374
C	0.000000	0.000000	-1.380750
H	0.000000	0.000000	2.449840
H	-2.121620	0.000000	1.224920
H	2.121620	0.000000	1.224920
H	-2.121620	0.000000	-1.224920
H	2.121620	0.000000	-1.224920
Cl	0.000000	0.000000	-3.057370

Molecule: Cyclohexanol

Atom	X	Y	Z
C	-1.239770	-0.114392	0.583142
C	1.193650	0.347302	0.581950
C	-0.065160	0.332812	-1.551100
C	1.258820	0.000000	-0.892162
C	-1.174600	-0.461692	-0.890970
C	0.084203	-0.447203	1.242080
H	-1.978980	-0.768359	1.023820
H	1.075120	1.410060	0.739408
H	-0.266608	1.394620	-1.534160
H	1.537650	-1.032350	-1.049700
H	-1.056080	-1.524450	-1.048430
H	0.285651	-1.509010	1.225140
H	-1.518600	0.917955	0.740679
H	2.121430	0.009614	1.021820
H	0.000292	-0.014801	-2.572460
H	1.998020	0.653968	-1.332840
H	-2.102390	-0.124004	-1.330840
H	-0.152638	0.929927	2.488560
O	0.000000	0.000000	2.556060

Molecule: Cyclopentane

Atom	X	Y	Z
C	1.266070	0.000000	0.000000
C	0.355743	1.180550	-0.311930
C	0.355743	-1.180550	-0.311930
C	-0.979482	0.767561	0.309729
C	-0.979482	-0.767561	0.309729
H	2.167290	0.000000	-0.585956
H	1.524380	0.000000	1.043470
H	0.731574	2.109890	0.076172
H	0.731574	-2.109890	0.076172
H	0.252220	1.271080	-1.378060
H	0.252220	-1.271080	-1.378060

H	-1.032440	1.135570	1.318350
H	-1.032440	-1.135570	1.318350
H	-1.806480	1.175110	-0.243016
H	-1.806480	-1.175110	-0.243016

Molecule: 2,3-difluorobutane

Atom	X	Y	Z
C	0.671074	-0.355409	0.674901
C	-0.671074	0.355409	0.674901
C	1.453540	0.040346	-0.565159
C	-1.453540	-0.040346	-0.565159
H	-0.513391	1.418740	0.674901
H	0.513391	-1.418740	0.674901
H	2.403500	-0.462765	-0.565159
H	-2.403500	0.462765	-0.565159
H	1.611220	1.103680	-0.565159
H	-1.611220	-1.103680	-0.565159
H	0.899716	-0.239766	-1.442860
H	-0.899716	0.239766	-1.442860
F	1.373770	0.000000	1.788540
F	-1.373770	0.000000	1.788540

Molecule: Formaldehyde

Atom	X	Y	Z
O	-0.003853	0.000000	0.668021
C	-0.000925	0.000000	-0.523312
H	0.953577	0.000000	-1.071270
H	-0.917208	0.000000	-1.133030

Molecule: N-methylformamide

Atom	X	Y	Z
C	1.008190	0.861231	0.000000
C	-0.655386	-0.858125	0.000000
H	1.277340	1.818200	0.000000
H	-0.958433	1.250440	0.000000
H	0.223231	-1.477460	0.000000
H	-1.240220	-1.065860	0.877704
H	-1.240220	-1.065860	-0.877704
O	1.848370	0.000000	0.000000
N	-0.262874	0.537450	0.000000

Molecule: Nitrobenzene

Atom	X	Y	Z
C	0.000000	0.000000	1.380750
C	-1.195760	0.000000	0.690374
C	1.195760	0.000000	0.690374
C	-1.195760	0.000000	-0.690374
C	1.195760	0.000000	-0.690374
C	0.000000	0.000000	-1.380750
H	0.000000	0.000000	2.449840
H	-2.121620	0.000000	1.224920
H	2.121620	0.000000	1.224920
H	-2.121620	0.000000	-1.224920
H	2.121620	0.000000	-1.224920
N	0.000000	0.000000	-2.851840
O	-1.070380	0.000000	-3.416780
O	1.070380	0.000000	-3.416780

Molecule: Propanamide

Atom	X	Y	Z
C	-0.644129	0.000000	0.120585
C	0.662738	0.000000	-0.613716
C	-0.384069	0.000000	1.616910
H	1.546320	0.000000	-2.403340
H	-0.178799	0.000000	-2.423490
H	-1.204740	0.877704	-0.145673
H	-1.204740	-0.877704	-0.145673
H	0.176546	0.877704	1.883170
H	0.176546	-0.877704	1.883170
H	-1.321230	0.000000	2.143480
O	1.697620	0.000000	0.000000
N	0.677944	0.000000	-1.915420

Molecule:Propanoic acid

Atom	X	Y	Z
C	-0.655468	0.000000	-0.102027
C	0.378474	0.000000	0.983358
C	0.032125	0.000000	-1.456220
H	0.653016	0.000000	2.955600
H	-1.269530	0.877704	-0.011951
H	-1.269530	-0.877704	-0.011951
H	0.646184	0.877704	-1.546300
H	0.646184	-0.877704	-1.546300
H	-0.709321	0.000000	-2.234550
O	1.547860	0.000000	0.700248
O	0.000000	0.000000	2.270090
